# Sociodemographic and behavioural differences between frequent and non-frequent users of convenience food in Germany

**DOI:** 10.3389/fnut.2024.1369137

**Published:** 2024-03-22

**Authors:** Anna Dittmann, Lea Werner, Lena Hörz, Theresa Luft, Fiona Finkbeiner, Stefan Storcksdieck genannt Bonsmann

**Affiliations:** Department of Nutritional Behaviour, Max Rubner-Institut (MRI)—Federal Research Institute of Nutrition and Food, Karlsruhe, Germany

**Keywords:** food consumption, convenience food, processed food, food reformulation, sociodemographic factors, behavioural factors, cooking skills

## Abstract

**Introduction:**

Convenience foods are a double-edged sword in that they provide quick and easy nutrition but may promote non-communicable diseases related to excess intakes of sugar, fat, and salt. To inform the German national reduction and innovation strategy for less sugar, fat, and salt in processed foods, the present study sought to analyse the consumption frequency of selected convenience foods and to determine sociodemographic and behavioural factors that characterise frequent users.

**Methods:**

In a representative computer-assisted telephone interview survey in the adult German population (*N* = 3,997) conducted in 2018, consumption frequency of 21 convenience foods was assessed. To characterise frequent in contrast to non-frequent users, data on sociodemographics and behavioural aspects were compared. Statistical analyses comprised chi-square tests with Bonferroni correction as well as Spearman’s rank correlation. Cramer’s V was used to determine the strength of an association.

**Results:**

Overall and among frequent users (7.7% of the sample) sweet convenience foods and savoury cooking aids were consumed most frequently. Around 75% of the participants indicated little-to-no consumption of 19 of the 21 convenience foods. Male gender (*p* < 0.001), younger age (*p* < 0.001), and not having a high level of education (*p* = 0.017) were identified as key characteristics of frequent users. Furthermore, frequent users were more likely than non-frequent users to live in a family household (*p* = 0.003) or without a partner (*p* < 0.001), and to work in shifts (*p =* 0.002). Additionally, they showed significantly lower cooking skills (*p* < 0.001).

**Conclusion:**

Public health interventions to limit excess intakes of sugar, fat, and salt from convenience food in Germany should target people of male gender, younger age, and having a lower level of education. On the behavioural side, developing the skills to cook from scratch emerged as major point of focus. Simultaneously, reformulation of the food offer should continue in order to help transition to a more health-promoting food environment.

## 1 Introduction

Convenience foods such as ready meals, instant soups and sweetened breakfast cereals make a sizable contribution to many people’s diets (1, 2), not least owing to their taste, being time-saving, often highly palatable and having a long shelf-life (1, 3). Convenience food is also popular among German consumers: based on data from the last major nutrition survey in 2005–2007, processed foods and drinks accounted for 53.1% of total energy intake (2). However, German market data show that convenience food may have high contents of energy, sugar, fat, and/or salt (4). High intakes of energy and these nutrients, in turn, increase the risk for developing obesity and noncommunicable diseases (5).

Reducing energy, sugar, fat, and salt contents through food reformulation is widely recognized as one means to improve the food environment and thus promote healthier food choices. In 2016, the European Union called on its member states to develop national strategies in this regard by the end of 2017 (6). In 2018, Germany initiated the “National Reduction and Innovation Strategy for Sugar, Fats, and Salt” (NRI) with the aim to improve the nutritional composition of processed food through reformulation by voluntary commitments of the food sector (7). Within this context, the German product monitoring was designed to collect information on energy and nutrient contents of convenience food on the German market from mandatory labelling (8).

Reformulation efforts should primarily address product groups that are most likely to contribute to high intakes. For this purpose, data on frequently consumed convenience foods in Germany is needed. Given the complexity of daily food work, food choices, and food environments (9–11), such data should be viewed in relation to sociodemographic characteristics and individual circumstances that promote frequent consumption. Previous studies from Europe, Australia and Brazil suggest that especially men (12–16), younger age groups (12–14, 17–22), and people with lower levels of education (15, 21) as well as low cooking skills (18, 23–25) tend to consume convenience food more frequently. Variables such as household size and composition (3, 15, 18, 26), daily schedules (11), or taste (27, 28) are also known to have an impact on food choice. For Germany, a few studies confirmed some of these associations (2, 29, 30). However, the existing literature (both for Germany and worldwide) mostly had a different focus, namely either on subgroups of convenience foods (mainly based on their degree of processing) (12–17, 19–21, 24, 25, 27, 30, 31) or on identifying subgroups of consumers by assigning them to different dietary or lifestyle patterns (2, 3, 22, 26, 29, 32).

Hence, the aim of this research was to investigate the consumption frequency of convenience foods, selected due to their potential relevance for public health, in a representative sample of the German adult population. Based on this information, the objective was to determine if there are individuals showing a particularly frequent consumption of a range of convenience foods, and if so, which sociodemographic and behavioural characteristics set them apart from non-frequent users.

## 2 Methods

### 2.1 Study design and population

For this cross-sectional study, a computer-assisted telephone interview (CATI) was conducted from January to March 2018. Representative sampling was based on the German census by age, gender, and region. The survey was conducted in randomly selected households using generated telephone numbers. Only respondents ≥18 years old, living in Germany and with sufficient knowledge of the German language were considered. Among the 4,000 adults who answered the questionnaire, 3 respondents were excluded due to implausible or incomplete data. The final sample comprised 3,997 subjects, characterised by an equal gender split and a mean age of 50.4 years (SD 17.5) (see Table 1, further details on the sample characteristics can be found in the Supplementary Table S1).

**Table 1:** Sociodemographics of the entire study sample (*N* = 3,997) of 18- to 80-year-old adults living in Germany in comparison to the German population.

Variable		Sample *N* = 3,997		German population^1^
		*n*	%	%
Gender	Male	1991	49.8	49.5
	Female	2006	50.2	50.5
Age (in years)	18–24	370	9.3	9.4
	25–34	608	15.2	16.1
	35–50	979	24.5	26.6
	51–64	1,099	27.5	27.2
	65–80	941	23.5	20.7
Educational level^2^	Low	709	17.7	13.3
	Medium	1,931	48.3	57.6
	High	1,135	28.4	29.1
	Not attributable	222	5.6	–
Employment	Full-time	1,526	38.2	59.2
	Part-time	602	15.1	
	Other (e.g., parental leave, marginally employed)	312	7.8	
	Non-employed (incl. students, pensioners)	1,474^3^	36.9^3^	40.8
	No indication	83	2.1	–
Household size	One-person	920	23.0	41.9
	Two-person	1,634	40.9	33.8
	Multi-person	1,426	35.7	24.4
	No indication	17	0.4	–
Household with children < 18 years	Yes	893	22.3	19.5
	No	3,061	76.6	80.5
	No indication	43	1.1	–

1 Based on microcensus 2018. The microcensus is an annual representative household survey conducted by the Federal Statistical Office of Germany, in which 1% of the entire population is surveyed on behalf of the entire population aged 15 years and older.

2 In this study, educational level is classified using CASMIN. In the microcensus, educational level is classified using ISCED2011 and data refer to the population aged 25–64 years.

3 Incl. pupils (*n* = 38, 1.0%) who were not posed the question.

### 2.2 Data collection

The questions asked were predominantly derived from the German National Nutrition Survey II (33). The questionnaire was programmed using the Software “Q.” Questions and answer specifications were either randomised or rotated. Data was collected in compliance with the General Data Protection Regulation. No sensitive data was collected. A pre-test with 30 interviews was conducted to test the questionnaire for practicability, comprehensibility, and completeness, and adjust it accordingly. The questionnaire can be found in Supplementary Material S1.

#### 2.2.1 Convenience food consumption

The selection of convenience foods covered in the study was based on own product monitoring data (4) on frequently purchased convenience foods and their energy and nutrient contents. As per household panel data from GfK (market research institute Growth from Knowledge) most of these convenience foods had a customer reach of over 50% (share of consumers buying the product at least once in a year) and/or a mean nutrient content above the mean contents of all monitored food groups (>11 g fat or > 6.2 g sugar or > 0.8 g salt per 100 g of product). Using this approach, convenience foods were selected that likely contribute to a high intake of fat, sugar, or salt due to their composition and household purchase quantity. In addition, foods with a high degree of convenience (e.g., frozen chips, instant mashed potato) or lacking the obviousness of an unfavourable nutrient content (e.g., sweetened yoghurt) were considered during the selection process. In total, 21 types of convenience food were included in the questionnaire (Table 2).

**Table 2:** Selected convenience foods, grouped as blocks as asked, and their description, where considered necessary for comprehension.

P1	Ready-mixed muesli
P2	Sweetened cornflakes or similar
P3	Sweetened yoghurt
P4	Ready-made desserts (chilled), e.g. pudding, mousse, rice pudding
P5	Ready-made pasta sauce
P6	Instant sauce powder; powder for the preparation of classic savoury sauces, e.g. gravy, sauce Hollandaise, herb or cream sauces
P7	Instant mashed potato / powder for making potato dumplings
P8	Stock cubes or powder; for the preparation of vegetable or meat broths
P9	Seasoning mixes for the quick preparation of meat, pasta or vegetable dishes
P10	Instant soup (in bags or cups)
P11	Plant based meat substitutes (PBMS); e.g. vegetarian patties, tofu sausages
P12	Frozen chips
P13	Frozen pizza
P14	Ready-made stew (with pulses or meat)
P15	Meat and sausage salad (chilled); savoury “salads” made with boiled sausage, onions, vinegar, oil, and sometimes pickles
P16	Potato salad (chilled)
P17	Vegetable salad and raw vegetable salad (chilled)
P18	Frozen pasta dishes, e.g. lasagne
P19	Filled Pasta (chilled or frozen), e.g. ravioli
P20	Frozen fish dishes; ready-prepared, frozen dishes made from fish or seafood, e.g. fish fingers
P21	Frozen meat dishes; ready-prepared chilled or frozen meat dishes, e.g. chicken fricassee, goulash, stir fried meat

Underlined words are used as terms for the respective food in the further text.

Participants were asked about the frequency of consumption at home of each convenience food in the past 12 months. In addition to the group of convenience foods P12–P21 (Table 2), which were queried under the term “ready meals”, the consumption frequency of ready meals in general, defined as P12–P21 plus further comparable products, was asked. For all convenience foods, consumption outside the home (e.g., in restaurants, canteens, or at a friend’s place) was explicitly excluded. Responses were recorded on a 6-point scale: “never”, “less often”, “1–2 times per month”, “approx. once per week”, “multiple times per week”, and “daily”. Additionally, the options “I do not know the product” and “no information/I do not know (how often)” were given. The response option “I do not know the product” was later assigned to the option “never”. For statistical reasons, the options were further aggregated, i.e., little-to-no consumption (sum of never, less often, and 1–2 times per month) was compared to regular consumption (sum of approx. once per week, multiple times per week, and daily).

In order to identify respondents with an overall frequent consumption of convenience food, a new variable “frequent users” was created. As no validated methodological approaches for the definition of frequent users could be derived from the literature, a data-driven definition was developed on the basis of an explorative procedure. The diversity of the surveyed convenience foods and their different consumption frequencies considered to be critical, were taken into account. At the same time, it was ensured that the group of frequent users was sufficiently large for further analysis. Accordingly, frequent users of convenience food were defined as respondents consuming at least 8 of the 21 convenience foods approx. once a week or more often, or at least 5 of the 21 convenience foods multiple times a week or daily. These cut-offs should not be seen as fixed values but as a judgment call based on nutritional expertise and statistical requirements.

#### 2.2.2 Sociodemographics

Sociodemographic variables include age, gender, region of residence, highest school-leaving certificate, highest level of vocational training, employment status (incl. shift work), number of persons in the household (incl. children), and monthly household net income. Based on the international classification system “Comparative Analyses of Social Mobility in Industrial Nations” (CASMIN), data on the highest school-leaving certificate and on vocational training were combined to reflect the level of education (categorised as low, medium, or high).

#### 2.2.3 Behavioural characteristics

Data on nutritional behaviour, such as dietary habits, shopping and cooking behaviour, meal intake at home, reasons for cooking from fresh ingredients, and reasons for using convenience food, were collected using mostly closed questions with simple choices or rating questions with a 5-point Likert scale (level of agreement: 1 = no agreement, 5 = full agreement).

To assess dietary habits, participants were asked which of the dietary practices listed they were familiar with, and if so, whether they practised them. Respondents who reported practising at least one of the diets in question were categorised as “following a special diet”.

### 2.3 Statistical analysis

The consumption frequency of single convenience foods was determined for the entire sample and stratified by gender and age group as well as for frequent vs. non-frequent users using cross-tabulations. To identify significant differences between respondent groups in terms of sociodemographic and behavioural variables as well as consumption frequency, chi-square tests with Bonferroni correction for multiple comparisons were conducted using SPSS Custom Tables. A *p*-value of <0.05 was considered statistically significant. The strength of an association was determined using Cramer’s V. To further explore associations between two rank variables (e.g., reasons for consumption of convenience food and consumption frequency of convenience foods), Spearman’s rank correlation analysis was performed. Correlations were considered meaningful if the correlation coefficient *r* was ≤ − 0.3 or ≥ 0.3 and statistically significant (*p* < 0.05). For the comparison of two Spearman’s rank correlation coefficients of independent samples (e.g., frequent vs. non-frequent users), Fisher’s z-transformation was used. All analyses were performed using SPSS Statistics for Windows, version 24.0 (IBM Corp., Armonk, NY, United States).

## 3 Results

### 3.1 Consumption frequency of convenience food among the study population

**Figure 1:**
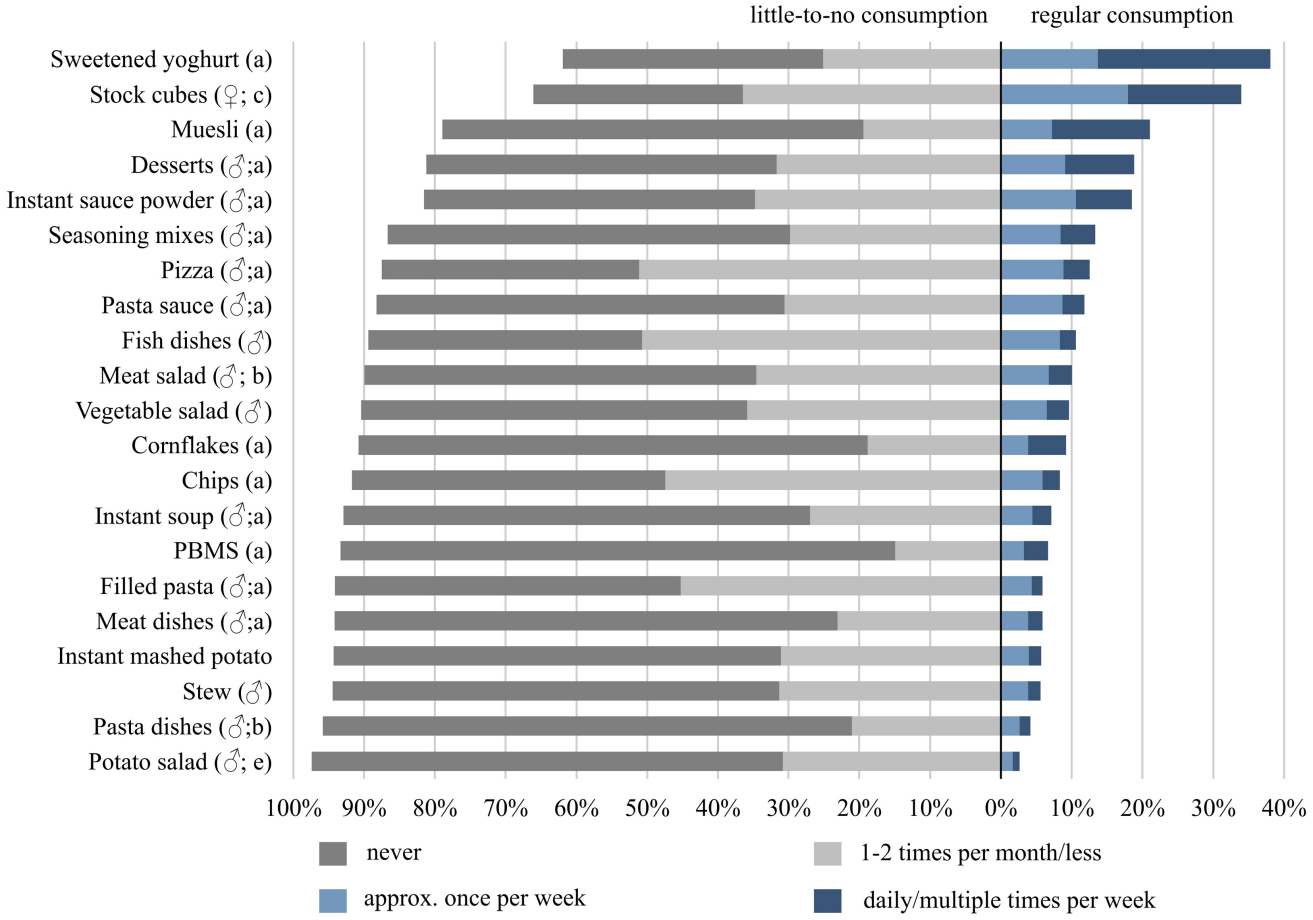
Consumption frequencies of the 21 selected convenience foods in the entire study sample of 18- to 80-year-old adults living in Germany (*N* = 3,997). Categorical variables were analysed by using the chi-squared test with Bonferroni post-hoc test for multiple comparisons and expressed as percentages. Percentage points missing to 100% correspond to “no information/I do not know (how often)”. ♂ indicates a significant difference between gender with higher share for men and ♀ for women. Letters a-e indicate significant differences between age groups, with the respective letter describing the highest share in the age group based on the following code: a = 18–24 years, b = 25–34 years, c = 35–50 years, d = 51–64 years, e = 65–80 years. Differences regarding little-to-no consumption and regular consumption (*p* < 0.05; Cramer’s V = 0.04–0.23).

Figure 1 shows the consumption frequency of the 21 convenience foods investigated in the total sample. The top 5 convenience foods consumed on a regular basis, i.e., at least approx. once a week, were: sweetened yoghurt (38.1%), stock cubes (34.0%), muesli (21.0%), desserts (18.8%), and instant sauce powder (18.5%).

Looking at the other end of the scale, three quarters or more reported little-to-no consumption for all convenience foods except sweetened yoghurt and stock cubes. Convenience foods with the largest shares of non-users were plant-based meat substitutes (PBMS) (78.4%), pasta dishes (74.8%), cornflakes (71.9%), and meat dishes (71.0%).

To see whether single convenience foods were consumed particularly frequently among specific gender or age groups, respective associations were tested. Significant gender differences regarding the consumption frequency were found for 15 of the 21 convenience foods (*p* < 0.05; Cramer’s V = 0.04–0.14). With the exception of stock cubes, the shares of respondents indicating regular consumption of convenience foods were higher among men than among women. Significant age differences were found for 17 of the 21 convenience foods (*p* < 0.05; Cramer’s V = 0.06–0.23): In most cases, there was a downward trend in the share of respondents reporting regular consumption from younger to older age groups, with the largest discrepancy seen for pizza [32.5% (18–24 years) vs. 5.4% (65–80 years); *p* < 0.001]. Figure 1 additionally indicates whether there were significant differences regarding gender and age for each convenience food, and points out the respective group with the highest share. Details can be found in the Supplementary Figures S1, S2.

With regard to ready meals in general, 11.1% of the study sample indicated no consumption of any ready meals in general. Overall 27% indicated regular consumption, i.e., at least approx. once a week. However, a quarter of these 27% did not regularly consume any of the single ready meals asked for (10 items). For the single ready meals, the overall shares for regular consumption ranged from 2.6% (potato salad) to 12.5% (pizza).

### 3.2 Comparison of frequent vs. non-frequent users of convenience food

Overall, 7.7% of the study sample qualified as frequent users. To determine sociodemographic and behavioural factors which could influence the consumption frequency of convenience food, frequent and non-frequent users are contrasted in the following.

#### 3.2.1 Consumption frequency of convenience food

**Figure 2:**
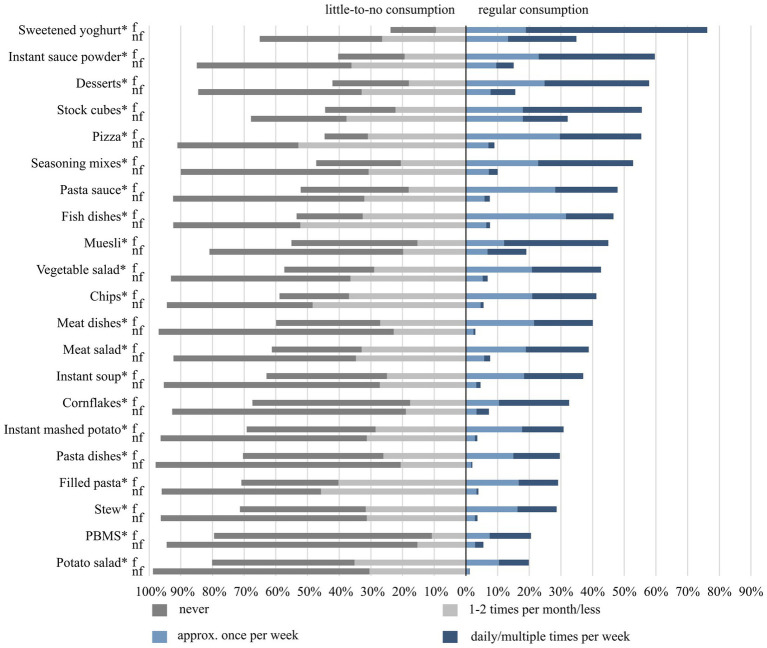
Consumption frequencies of the 21 selected convenience foods among frequent (f) and non-frequent users (nf) of the study sample of 18- to 80-year-old adults living in Germany. Categorical variables were analysed by using the chi-squared test with Bonferroni post-hoc test for multiple comparisons and expressed as percentages, stratified by frequent (*n* = 307) and non-frequent users (*n* = 3,690). Percentage points missing to 100% correspond to “no information/I do not know (how often)”. *indicates significant differences between frequent and non-frequent users regarding little-to-no consumption and regular consumption (*p* < 0.001; Cramer’s V = 0.17–0.45).

Figure 2 illustrates the consumption frequency of convenience foods among frequent and non-frequent users. The convenience foods consumed on regular basis by at least 50% of frequent users were sweetened yoghurt, instant sauce powder, desserts, stock cubes, pizza, and seasoning mixes. Potato salad and PBMS were the least consumed convenience foods by frequent users – less than 21% indicated their regular consumption.

Compared to non-frequent users, significant differences in the consumption frequency were found for all convenience foods, with the shares of respondents indicating regular consumption of the selected convenience foods being higher among frequent users than among non-frequent users (*p* < 0.001 for all; Cramer’s V = 0.17–0.45).

Looking at the consumption frequency of ready meals in general, almost 75% of frequent users indicated regular consumption, whereas the remaining quarter indicated little-to-no such consumption.

#### 3.2.2 Sociodemographic characteristics

The sociodemographic characteristics of frequent and non-frequent users are shown in Table 3. While men and women were evenly distributed among non-frequent users, men were more prevalent among frequent users. Looking at the distribution across age groups, there was a shift towards younger ages among frequent users, paralleled by a shift to older ages among non-frequent users. Furthermore, the share of respondents with a high level of education was lower among frequent users. Regarding the employment status, the share of respondents indicating “other”, e.g., being on parental leave, marginally employed, or “working in shifts” was higher among frequent than among non-frequent users. In terms of living conditions, frequent users were more likely to report living in multi-person households and having children living in their household than non-frequent users. Although more than three-quarters of frequent users indicated living together with a partner, this was a lower share than among non-frequent users. Regarding household net income, there were no significant differences between frequent and non-frequent users. Notably, more than 25% chose not to divulge information about their income. Concerning dietary habits, 31.9% of frequent users stated following a special diet in general. No significant differences were found between frequent and non-frequent users, except for higher shares of frequent users indicating following a halal [8.5% (*n* = 10) vs. 2.0% (*n* = 25); *p <* 0.001; Cramer’s V = 0.12] or raw food diet [7.1% (*n* = 18) vs. 3.1% (*n* = 102); *p* = 0.001; Cramer’s V = 0.06]. These differences should be treated with caution due to small subgroup sizes.

**Table 3:** Sociodemographic characteristics of frequent (f) and non-frequent users (nf) among 18- to 80-year-old adults living in Germany.

Variable		Frequent users (*n* = 307; 7.7%)	Non-frequent users (*n* = 3,690; 92.3%)
		%	%
Gender	Male*	65.1	48.5
	Female*	34.9	51.5
Age (in years)	18–24*	18.6	8.5
	25–34*	19.5	14.9
	35–50	20.5	24.8
	51–64*	22.5	27.9
	65–80*	18.9	23.9
Educational level	Low	19.5	17.6
	Medium	49.5	48.2
	High*	21.5	29.0
	Not attributable*	9.4	5.2
Employment	Full-time	35.5	38.4
	Part-time	14.7	15.1
	Other (e.g., parental leave, marginally employed)*	14.0	7.3
	Non-employed (incl. students, pensioners)	32.2^1^	37.3^1^
	No indication	3.6	2.0
Shift work (*n* = 174/2,113)^2^	Yes*	29.3	19.4
	No*	69.0	80.3
	No indication	1.7	0.3
Household size	One-person	21.8	23.1
	Two-person*	33.2	41.5
	Multi-person*	44.0	35.0
	No indication	1.0	0.4
Living with a partner (*n* = 226/2,751)^2^	Yes*	76.1	86.8
	No*	23.5	12.8
	No indication	0.4	0.4
Household with children <18 years	Yes*	28.7	21.8
	No*	68.4	77.3
	No indication	2.9	0.9
Household net income (in €)	Less than 1,500	15.3	10.8
	1,500–2,000	12.1	11.6
	2,000–2,500	11.7	10.5
	2,500–3,000	8.8	9.7
	3,000–3,500	10.1	9.5
	3,500–4,000	5.5	7.2
	4,000–4,500	2.9	4.2
	4,500 and more	8.1	8.8
	No indication	25.4	27.8
Special diet^3^	Yes	31.9	28.5
	No	68.1	71.5

1 Incl. pupils (2.0% among frequent users resp. 0.9% among non-frequent users) who were not posed the question.

2 Sample size differs, as not all respondents were posed all questions, due to filter variables (sample size of frequent/non-frequent users).

3 Includes vegan/vegetarian diet, predominantly plant-based diet, raw food diet, paleo diet, food combining diet, low-carb diet, salt-reduced diet, lactose-free/reduced diet, gluten-free/reduced diet, reduction diet, kosher diet, halal diet. *indicates significant differences between frequent and non-frequent users (*p* < 0.05).

#### 3.2.3 Behavioural characteristics

**Figure 3:**
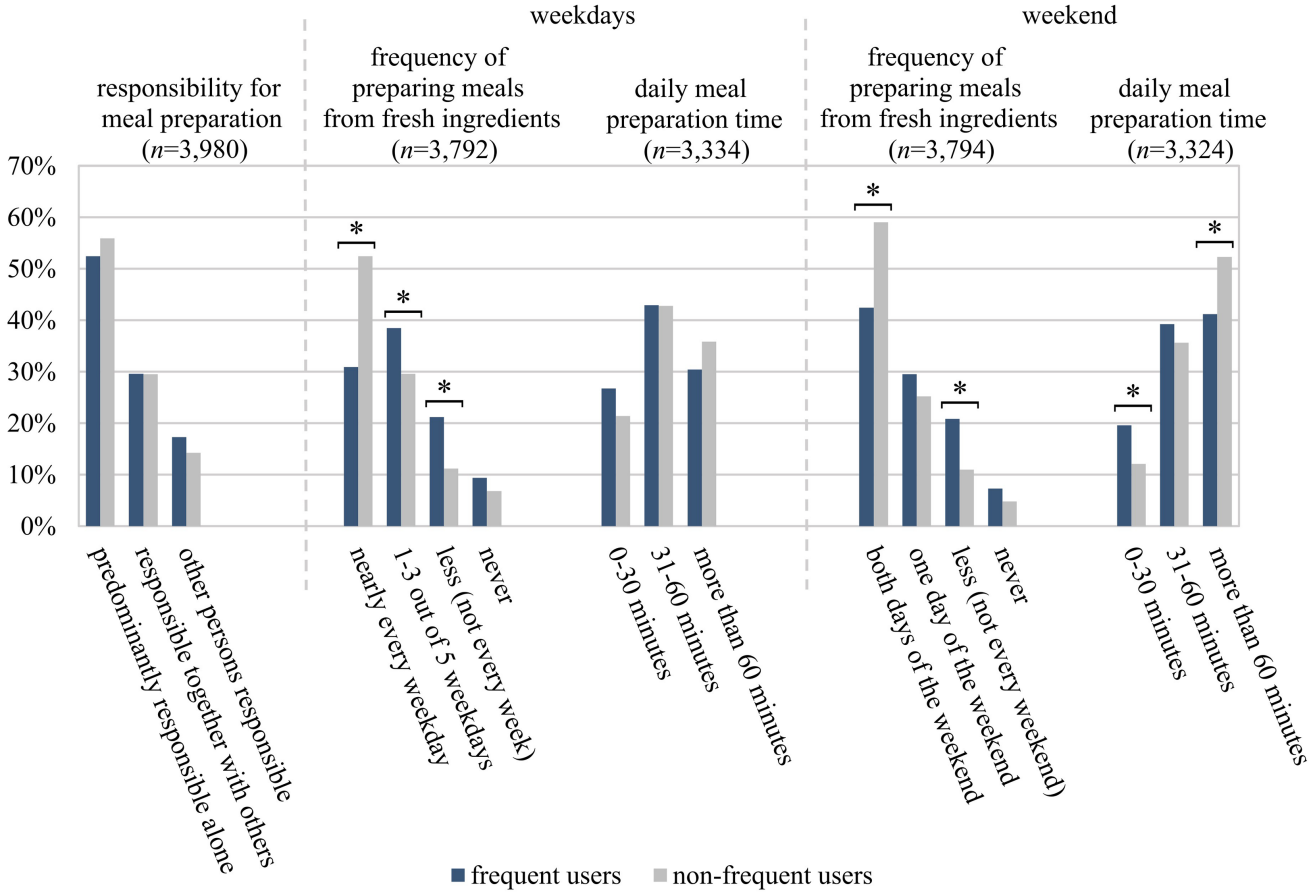
Cooking behaviour of frequent (f) and non-frequent users (nf) of the study sample of 18- to 80-year-old adults living in Germany. Categorical variables were analysed by using the chi-squared test with Bonferroni post-hoc test for multiple comparisons and expressed as percentages, stratified by frequent and non-frequent users (*n* = 3,324–3,980). Sample sizes differ, as questions did not apply to all respondents (*n* = 595 and *n* = 193 resp.) or individuals did not provide any information (*n* = 10–78). *indicates significant differences between frequent and non-frequent users (*p* < 0.05).

Regarding the general nutritional behaviour, frequent and non-frequent users differed only in some aspects. Approx. 84% of frequent users said they were involved in deciding what food to buy, which was significantly lower than the share of nearly 90% among non-frequent users (*p* = 0.004; Cramer’s V = 0.05). Regarding the self-assessment of cooking skills, 32% of frequent users indicated medium and 25% low cooking skills, compared to 25% and 12% of non-frequent users, respectively (medium *p* = 0.010, low *p* < 0.001; Cramer’s V = 0.13). Regardless of whether they were frequent or non-frequent users, those reporting higher cooking skills also reported preparing meals from fresh ingredients more often during the week [(f) *r* = 0.4; (nf) *r* = 0.37] and at weekends [(f) *r* = 0.35; (nf) *r* = 0.34]. In general, respondents more often indicated cooking from fresh ingredients and taking more time to prepare meals at the weekend than during the week. For both weekdays and weekends, the shares of frequent users reporting cooking from fresh ingredients nearly every day was significantly lower than among non-frequent users (31% vs. 52%, *p* < 0.001 on weekdays; Cramer’s V = 0.12; 42% vs. 59%, *p* < 0.001 at weekends; Cramer’s V = 0.10) (Figure 3). Regarding meal preparation time, the responses of frequent and non-frequent users only differed significantly for the weekend: 52% of non-frequent users reported spending more than 60 min per day cooking at weekends, whereas the share of frequent users doing so was 41% (*p* = 0.001; Cramer’s V = 0.07) (Figure 3). Regardless of whether they were frequent or non-frequent users, those who reported preparing meals from fresh ingredients more often said they spent more time on meal preparation at weekends [(f) *r* = 0.37; (nf) *r* = 0.33].

**Figure 4:**
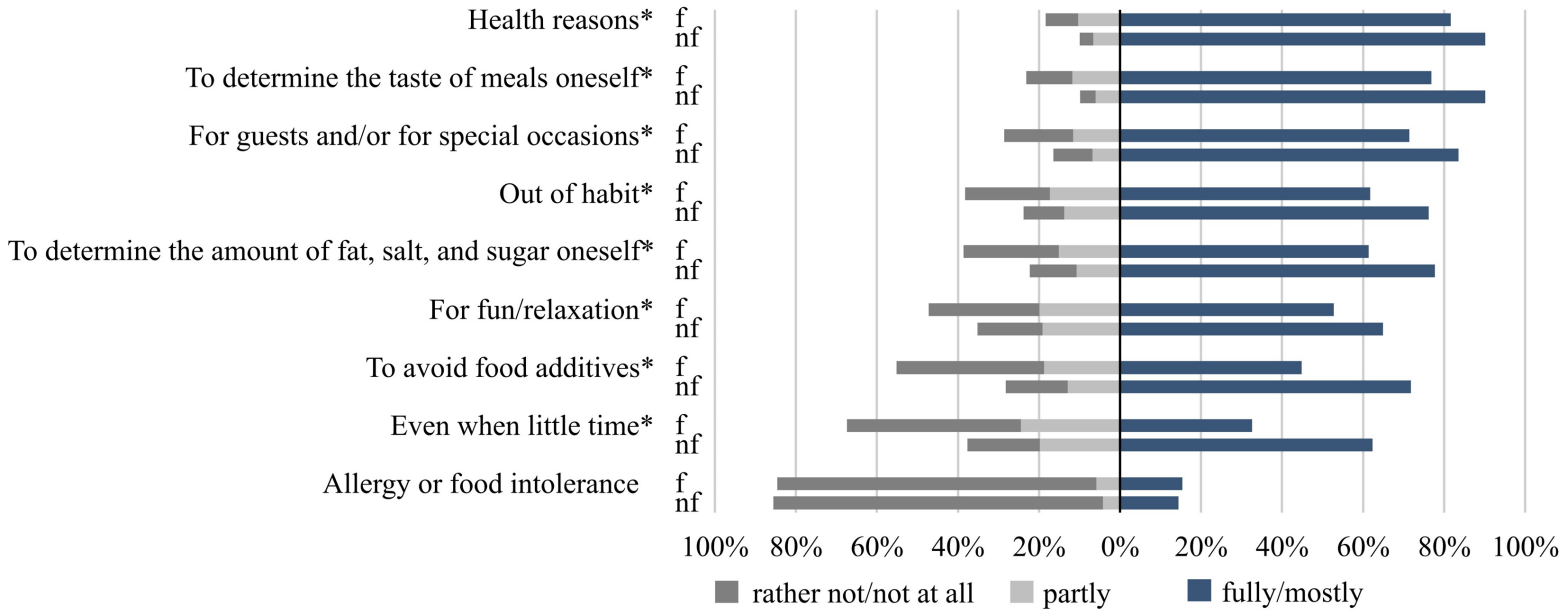
Agreement with reasons for cooking from fresh ingredients among frequent (f) and non-frequent users (nf) of the study sample of 18- to 80-year-old adults living in Germany. Categorical variables were analysed by using the chi-squared test with Bonferroni post-hoc test for multiple comparisons and expressed as percentages, stratified by frequent and non-frequent users (*n* = 3,634–3,657). Sample sizes differ, as questions did not apply to all respondents (*n* = 323) or individuals did not provide any information (*n* = 10–40). *indicates significant differences between frequent and non-frequent users (*p* < 0.05; Cramer’s V = 0.08–0.18).

Concerning reasons for cooking from fresh ingredients, Figure 4 shows that the shares of agreement with all statements except “allergy or food intolerance” were lower for frequent than for non-frequent users (*p* < 0.001 each; Cramer’s V = 0.08–0.18). This is particularly true for the statements “even when little time” and “to avoid food additives”. However, for the majority of reasons, more than 60% of both frequent and non-frequent users mostly or fully agreed with the statements.

**Figure 5:**
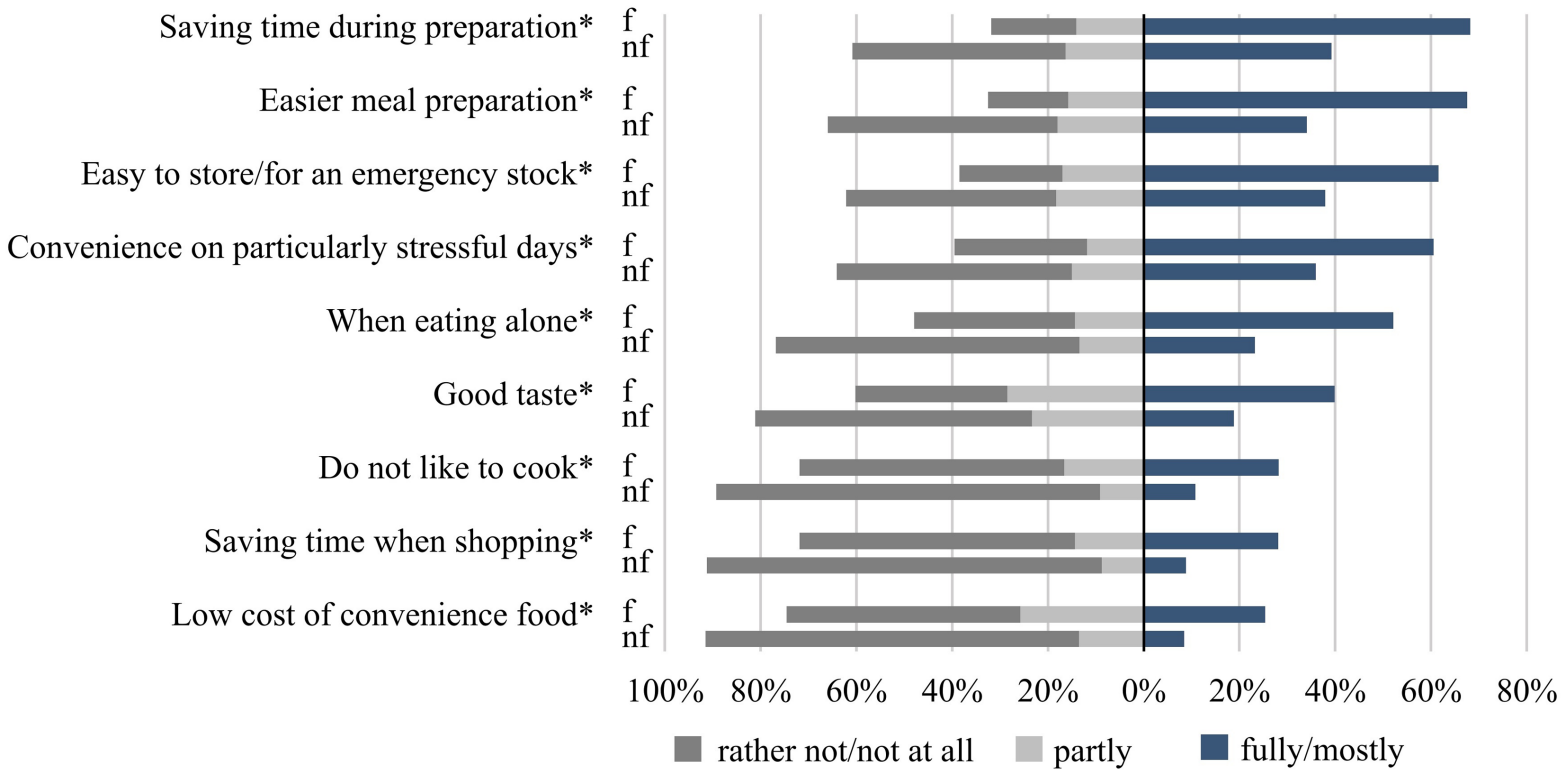
Agreement with reasons for consuming convenience food among frequent (f) and non-frequent users (nf) of the study sample of 18- to 80-year-old adults living in Germany. Categorical variables were analysed by using the chi-squared test with Bonferroni post-hoc test for multiple comparisons and expressed as percentages, stratified by frequent and non-frequent users (*n* = 3,811–3,912). Sample sizes differ, as questions did not apply to all respondents (*n* = 134–160) or individuals did not provide any information (*n* = 25–47). *indicates significant differences between frequent and non-frequent users (*p* < 0.05; Cramer’s V = 0.14–0.20).

Respondents’ agreement with statements concerning reasons for using convenience food is shown in Figure 5. Frequent users were more likely to agree with the statements than non-frequent users (*p* < 0.001 each; Cramer’s V = 0.14–0.20). The main reasons for using convenience food were related to convenience with regards to meal preparation or to storability/shelf-life (Figure 5). Less relevant reasons for using convenience food were “low cost of convenience food”, “saving time when shopping”, and “not liking to cook”. Those respondents reporting to use convenience food because they do not like to cook were more likely to report that their cooking skills were poor [(f) *r* = −0.39; (nf) *r* = −0.33], with a significantly stronger correlation found for frequent users.

Looking at correlations between reasons for use of convenience food and consumption frequency of the queried convenience foods, meaningful correlations were found for non-frequent users rather than for frequent users. E.g., non-frequent users reporting to use convenience food because it is convenient on particularly stressful days or for saving time during preparation were more likely to report regular consumption of pizza [(nf) *r* = 0.32 resp. 0.34]. Correlations with consumption frequency of seasoning mixes were found for reasons such as easier meal preparation, when eating alone, and good taste [(nf) *r* = 0.30 each]. The latter two were also found to be associated with the consumption frequency of pasta dishes [(nf) *r* = 0.33 resp. 0.32]. Low cost of convenience food as a reason was associated with the consumption frequency of pasta dishes [(nf) *r* = 0.32] and meat dishes [(nf) *r* = 0.30].

No striking results were found for other behavioural variables, such as meal intake at home and food shopping frequency (see Supplementary Figures S3, S4 for details).

## 4 Discussion

This representative study details the sociodemographic and behavioural characteristics of frequent users of a range of convenience foods in Germany in comparison to non-frequent users. These results are important to understand which consumer groups are most at risk of high intakes of sugar, fat, and salt from convenience food, and to derive targets for respective public health interventions.

### 4.1 General consumption frequency of convenience food

Looking at the study population as a whole, regular consumption (approx. once per week or more often) ranged from 38.1% (sweetened yoghurt) to 2.6% (potato salad) at the level of single convenience foods. Sweet foods (namely sweetened yoghurt, desserts, and muesli) as well as savoury product aids for cooking (especially stock cubes and instant sauce powder) were consumed far more regularly than foods which serve as main dishes such as meat dishes, pasta dishes, or stew. Among the main dishes, pizza was the convenience food most frequently consumed (12.5%), a result that mirrors its general popularity (34) and is in line with ready meal sales being highest for pizza in Germany (data 2016–2018) (35). Overall, higher shares were found for ready meals in general than for single ready meals, which is not surprising, as several products have been combined in this question. The fact that a quarter of respondents who stated that they regularly consumed ready meals in general did not report regular consumption of any of the single ready meals surveyed, might indicate that not all ready meals of relevance for respondents were covered. Another explanation might be that consumers with regular consumption of ready meals vary strongly in their product selection. Accordingly, they possibly alternate between several ready meals queried in the present study and beyond instead of consuming a few single ready meals particularly frequently. However, no conclusion can be drawn about the type of products referred to in this regard. Hence, questions on specific items may have higher validity than general questions that may lead to misjudgements of usage, as noted previously by Brunner et al. (18).

In general, studies on the consumption frequency of convenience food are scarce, and what can be found differs more or less in methods, scales, or foods included. Nonetheless, comparable results by others also indicate an irregular consumption of single savoury (main) dishes (18, 30). Unfortunately, those studies did not capture other convenience foods with a particularly high consumption in Germany, such as stock cubes or muesli.

An explanation for the different consumption frequencies found in the present study could be that the analysis comprised foods that are typically consumed on different occasions, e.g., for breakfast out of habit, such as muesli, or as a main dish for lunch or dinner where more variation might be sought (36). However, for some convenience foods, especially for main dishes, a more frequent consumption might have been expected, since one of the criteria for product selection in this study was a high customer reach as indicated by the GfK data (4). On the one hand, these discrepancies could be taken to indicate the presence of social desirability bias, which is not unusual for questionnaires (37) and which needs to be factored into all following interpretations. On the other, customer reach was computed as the share of households purchasing from a given product category at least once a year, whereas in the present study regular consumption was defined as approx. once a week or more. Therefore, an infrequent consumption as defined in the present study is perfectly compatible with a wide customer reach as per the GfK household panel data.

### 4.2 Frequent users of convenience food

For the entire sample, it is particularly striking that three quarters and more of respondents indicated little-to-no consumption for 19 of the 21 convenience foods investigated. Therefore, identifying and describing frequent users in comparison to non-frequent users is a promising approach to gain more robust insights into those parts of the population most at risk of high intakes of sugar, fat, and salt from convenience food. Defining frequent users as those consuming at least 8 of the 21 convenience foods approx. once a week or more often, or at least 5 of the 21 convenience foods multiple times a week or daily, 7.7% of all respondents in the present study qualified as frequent users. Two Brazilian studies defined frequent users as those who consumed at least 5 out of 10, respectively 13 convenience foods on the same day and computed shares of 14.3% and 18.2%. Those shares might be truly higher as such and/or higher because of the more aggregated food groups, comprising comparatively divergent and simply more foods (for example also bread, soft drinks, and margarine) (12, 31). However, the observed share of frequent users in the present study might be lower than the true share due to a possible social desirability bias, as frequent consumption of a wide range of convenience foods might be considered less socially accepted.

Given that there is almost no research based on a comparable methodology of frequent consumption of convenience food, results are also contrasted with studies focussing on the consumption of foods of different processing grades (e.g., ultra-processed vs. minimally processed foods according to the NOVA classification), food patterns identified statistically, or consumer-centred approaches such as food lifestyles.

In line with our observation, several other studies reported convenience food consumption to be more prominent among men (2, 12–16, 29), younger adults (2, 12–14, 17–22), and people with a lower educational background (15, 21). All three variables have been described already in connection to lower cooking skills (38, 39). Furthermore, lower cooking skills and higher consumption of convenience food seem to be connected, as shown in the literature (18, 23–25) and also in our study. Some studies reported an inverse association between income level and consuming convenience food (21, 40, 41). No such association was found in the present study, but our data could be limited in this regard, given that approx. 25% of respondents chose not to indicate their income bracket.

Consistent with the present findings, other studies reported more frequent convenience food consumption in people who lived in households with children (29), in multi-person households (3), or single (26, 29). For households with children, the literature is inconsistent in that there are also studies that show either a lower consumption (18) or no association regarding convenience food consumption (25). Additionally, results may differ according to number (15) and age of the children in the household (29). An explanation for this inconsistency may be that working parents, on the one hand, are willing to care for their children through cooking healthy meals from scratch, but are, on the other hand, under stress in arranging meals and duties (42). Older children may even demand certain convenience foods, such as chips (29). As reported by Moran et al. (9) such demands may also be an explanation for why lower-income families buy convenience food, knowing it will not be a waste of money and is easy to store to serve as a backup at the month’s end, if money runs short.

Among frequent users there were significantly more respondents who worked in shifts than among non-frequent users. A study with UK police officers pointed out that shift work promoted unfavourable eating habits by changing typical meal times, not eating with others, and using short-cuts in food preparation due to higher stress levels or less energy left for food work (43). The higher level of agreement of frequent users with “when eating alone” as a reason for using convenience food is notable. However, in addition to the factors assessed here, there might be other factors influencing the consumption frequency of certain (convenience) foods, such as food preferences (of consumers themselves or household members) (9, 28) or product marketing (9), which were outside the scope of this study.

Looking at the reasons for using convenience food, frequent users displayed an overall higher agreement than non-frequent users. Their agreement was highest for the following aspects: convenience, saving time, and easy storage/emergency stock. Accordingly, frequent users spent comparatively less time on cooking. Costa et al. (34) qualitatively identified similar reasons and pointed out that individuals might rely on convenience food as time-savers in order to achieve a better work-life-balance. In contrast, Brunner et al. (18) propose that habitualization rather than time is the most relevant variable in this regard, at least in the long term. The overall higher agreement of frequent users with the reasons given and the fact that good taste was far more relevant for them than for non-frequent users, could also be interpreted this way. Yet another explanation, proposed by Candel (26), is that individuals with a high consumption of convenience food are simply not that interested in daily food work. Our results concerning the comparatively lower involvement of frequent users in purchasing decisions, but also the fact that less cooking time was spent and fresh ingredients were used less often, can be interpreted this way. Last, results on food lifestyles suggest that there might be more than one cluster of people with specific characteristics who could be classified as frequent consumers, such as “kitchen evaders”, “convenience seeking grazers”, or “casual consumers” (3, 32).

Regardless of the frequency of convenience food consumption, there is still plenty of opportunity to use unprocessed or fresh ingredients. This is reflected in the relatively high shares of 60% or more of frequent users who agreed with 5 of 9 statements concerning reasons for using fresh ingredients. In addition, the share of frequent users who indicated preparing meals with fresh ingredients nearly every weekday was rather high at 31%. These results are attributable to the chosen definition of frequent users that applies to all types of convenience food (including, e.g., cooking aids and sweet products) covered in the present study as well as the set frequency of at least 8 products once per week. Looking at the convenience foods with the highest shares of regular consumption, there might be frequent users consuming mainly sweet products serving as a breakfast, snack or dessert and/or cooking aids for preparing meals from fresh ingredients rather than convenience foods intended as main dishes. This assumption is in line with the results of the additional question regarding ready meals in general. Around 25% of frequent users stated that they consumed ready meals in general 1–2 times per month or less, suggesting that these users consume other convenience foods than ready meals frequently. In contrast, the majority of frequent users indicated regular consumption of ready meals in general, implying that they strongly rely on these dishes. This heterogeneity supports the aforementioned suggestion that there might be several subgroups of frequent users (3, 32). As a next step, it is therefore planned to analyse convenience food consumption patterns to understand if there are specific clusters of convenience foods that would be suitable for targeted reformulation measures, and to identify consumer groups that would benefit most from these. Regardless of this, data from the German product monitoring indicate that there are already options with lower contents of sugar, fat, and salt on the market, no matter what type of convenience food [e.g., (44, 45)].

### 4.3 Strengths and limitations

Major strengths of the present study are the representative sample and the large sample size. Although, the sample is not representative in terms of socio-economic characteristics. Furthermore, the selection of convenience foods relevant to German consumers is based on purchasing statistics and information on the nutritional composition of such products on the German market.

The used data was self-reported and subjective and, as already mentioned, potentially biased by social desirability. The latter could be particularly true for data being collected with CATI due to the presence of an interviewer (46). Resulting answers might yield a more positive rating compared to other modes of data collection, at least for questions concerning mental well-being or health-related behaviour (47, 48). There may be an underreporting in consumption frequency of certain foods and an overreporting regarding cooking from scratch. It is known that questions concerning cooking skills mirror self-assurance instead of objective skills (49). Apart from this, capturing the consumption frequency of certain foods by retrospective questions builds on the ability to correctly recall and estimate general nutritional behaviour, is semi-quantitative at best and does not cover the entire diet (50). Also, out-of-home consumption was not considered. Another aspect that must be considered in any interviewer-based surveys is that interviewers must be well trained in order to be able to resolve interviewee queries about the meaning of questions. In the present study, efforts were made to limit this issue by involving nutritionists in the training of interviewers and providing them with a comprehensive handbook. The above notwithstanding, our data provide a good picture of who the critical consumer groups of frequently consumed convenience foods are in Germany.

## 5 Conclusion

Our study identified male gender, younger age, and having a lower level of education as key characteristics of frequent users of convenience food in Germany. Compared to others, those population groups would benefit most from food reformulation efforts. Additionally, targeted education campaigns, e.g., for families or shift workers, should focus on highlighting the availability of convenience foods with lower sugar, fat, and salt on the German market. Furthermore, low-threshold programs aiming at frequent users appear warranted to increase their appreciation of fresh food over convenience food as well as to improve their cooking skills. At the same time, reformulation of the convenience food offer should continue to support making the healthy choice the easy choice.

## Data availability statement

The raw data supporting the conclusions of this article will be made available by the authors, without undue reservation.

## Ethics statement

Ethical approval was not required for the studies involving humans because no sensitive data was collected. Respondents were informed in detail about the study objectives and interview procedures as well as the handling of data records and analyses, which was carried out anonymised. The studies were conducted in accordance with the local legislation and institutional requirements. Written informed consent for participation was not required from the participants or the participants’ legal guardians/next of kin in accordance with the national legislation and institutional requirements because verbal consent was obtained, as this was a telephone interview. It was made clear that participation was on a voluntary basis and could be terminated at any time.

## Author contributions

AD: Conceptualization, Formal analysis, Writing – original draft, Writing – review & editing, Methodology, Visualization. LW: Writing – original draft, Writing – review & editing. LH: Formal analysis, Methodology, Visualization, Writing – review & editing. TL: Writing – original draft, Writing – review & editing. FF: Writing – original draft, Writing – review & editing. SSgB: Conceptualization, Supervision, Writing – review & editing.
