# Supplementary material for: Sociodemographic and behavioural differences between frequent and non-frequent users of convenience food in Germany
Source: Front Nutr. 2024 Mar 22;11:1369137. doi: 10.3389/fnut.2024.1369137 (PMC10997035; doi:10.3389/fnut.2024.1369137)
Supplement: Supplementary file 1 [file Data_Sheet_1.zip › Supplementary Image S2.pdf]

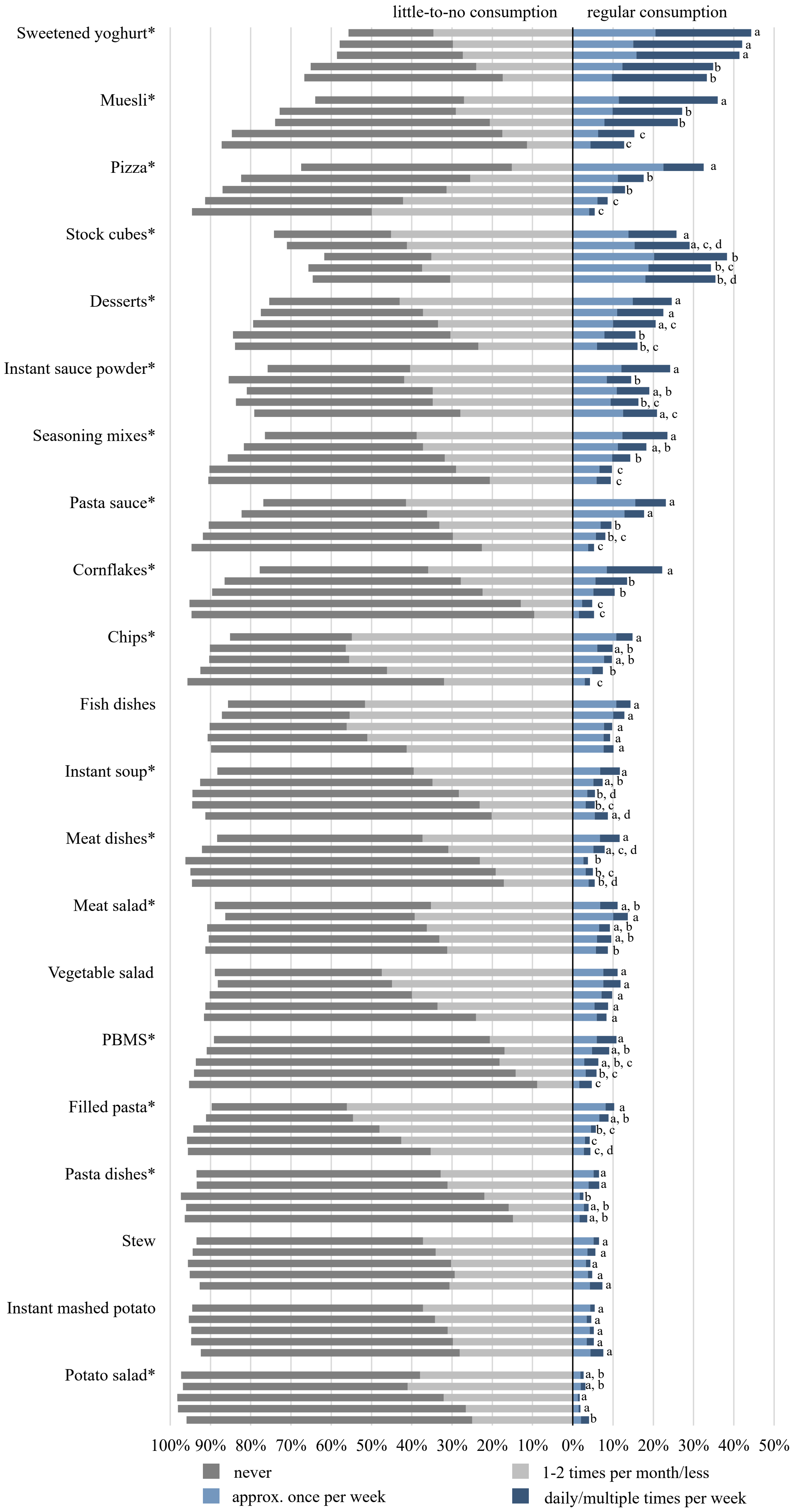

Supplementary Figure S2. Consumption frequencies of the 21 selected convenience foods in the entire study sample of 18- to 80-year-old adults living in Germany (*N* = 3,997) by age. Order of the five bars always according to increasing age-groups: 18-24 y., 25-34 y., 35-50 y., 51-64 y., 65-80 y. Categorical variables were analysed by using the chi-squared test with Bonferroni post-hoc test for multiple comparisons ($p < 0.05$) and expressed as percentages. Percentage points missing to 100% correspond to "no information/I do not know (how often)". *indicates significant differences between age groups regarding little-to-no consumption and regular consumption ($p < 0.05$; Cramerôs V=0.06-0.23); age-groups with non-identical letters differ at $p < 0.05$.
