# Supplementary material for: Sociodemographic and behavioural differences between frequent and non-frequent users of convenience food in Germany
Source: Front Nutr. 2024 Mar 22;11:1369137. doi: 10.3389/fnut.2024.1369137 (PMC10997035; doi:10.3389/fnut.2024.1369137)
Supplement: Supplementary file 1 [file Data_Sheet_1.zip › Supplementary Image S3.pdf]

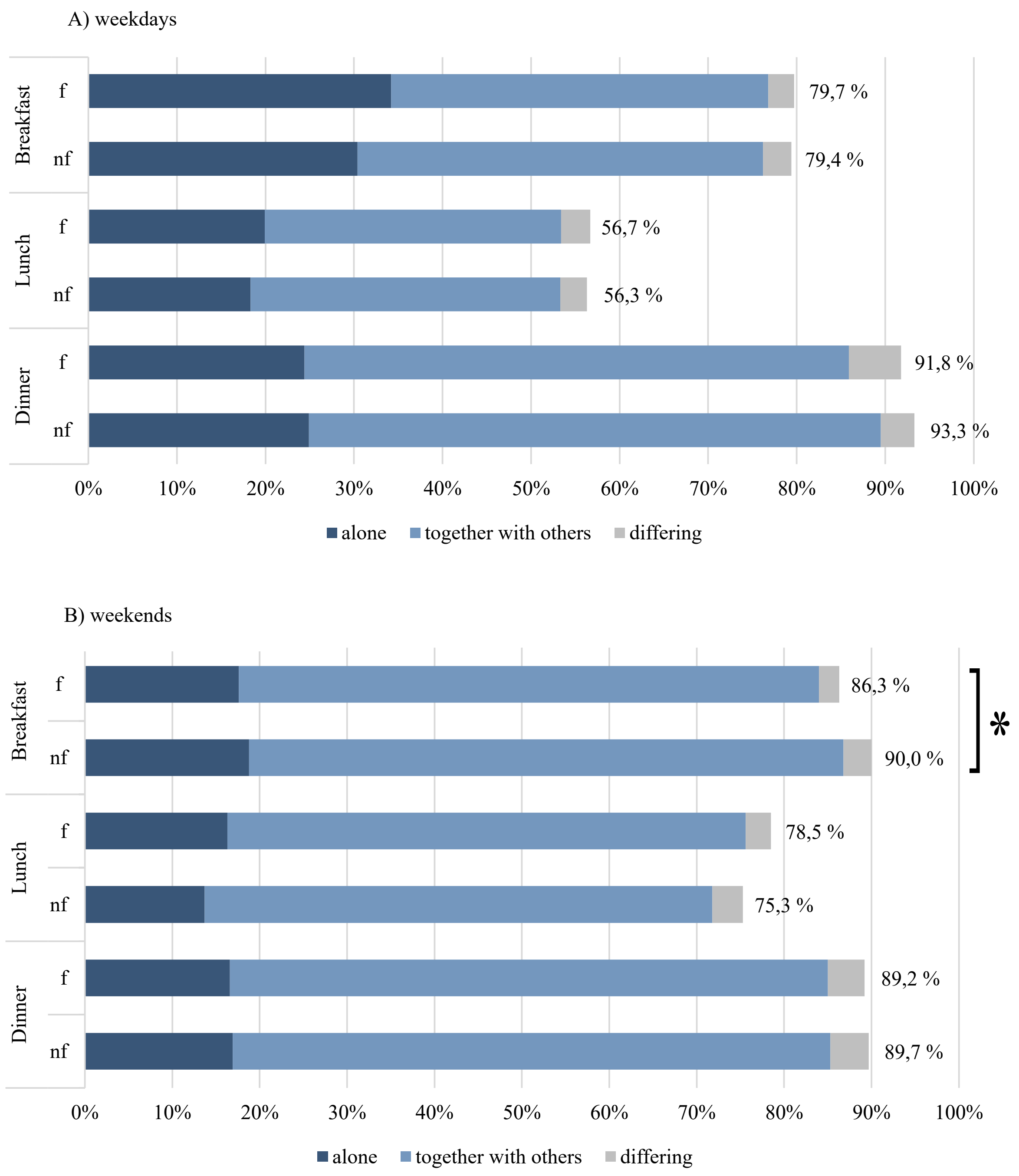

Supplementary Figure S3. Meal intake at home on weekdays (A) and at weekends (B) of frequent (f) and non-frequent (nf) users of convenience foods of the study sample of 18- to 80-year-old adults living in Germany.
Categorical variables were analysed by using the chi-squared test with Bonferroni post-hoc test for multiple comparisons ($p < 0.05$) and expressed as percentages stratified by frequent (n=307) and non-frequent users (n=3,690). *indicates significant differences between frequent and non-frequent users ($p < 0.05$).
