# Supplementary material for: Sociodemographic and behavioural differences between frequent and non-frequent users of convenience food in Germany
Source: Front Nutr. 2024 Mar 22;11:1369137. doi: 10.3389/fnut.2024.1369137 (PMC10997035; doi:10.3389/fnut.2024.1369137)
Supplement: Supplementary file 1 [file Data_Sheet_1.zip › Supplementary Image S4.pdf]

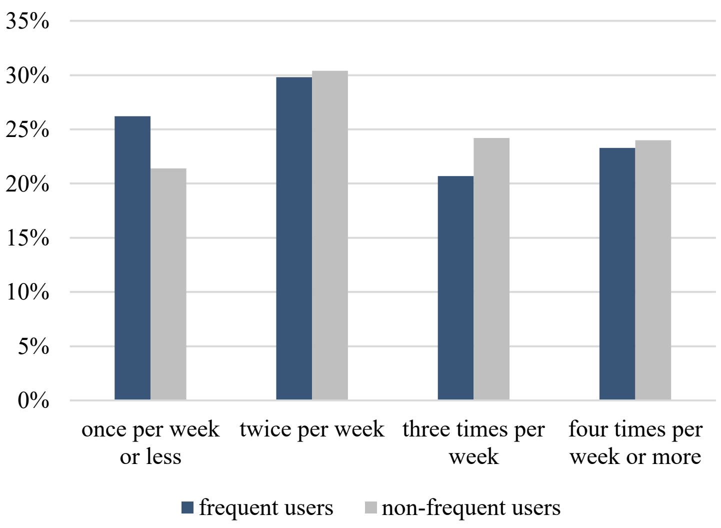

Supplementary Figure S4. Food shopping frequency of frequent and non-frequent users of convenience foods of the study sample of 18- to 80-year-old adults living in Germany. Categorical variables were analysed by using the chi- squared test with Bonferroni post-hoc test for multiple comparisons ($p < 0.05$) and expressed as percentages stratified by frequent and non-frequent users (n=3,970 as 27 individuals did not provide any information).
