# Supplementary material for: Sociodemographic and behavioural differences between frequent and non-frequent users of convenience food in Germany
Source: Front Nutr. 2024 Mar 22;11:1369137. doi: 10.3389/fnut.2024.1369137 (PMC10997035; doi:10.3389/fnut.2024.1369137)
Supplement: Supplementary file 1 [file Data_Sheet_1.zip › Supplementary Material 1.pdf]

Telefonbefragung zum Konsum vorgefertigter Produkte

Forschungsprojekt

172712

Fragebogennummer

Otto-Lilienthal-Straße 15
49134 Wallenhorst
Telefon +49 (0) 54 07 885-0

Telefonbefragung zum Konsum vorgefertigter Produkte
– Dezember 2017 –

Guten Tag, mein Name ist …
Ich bin Mitarbeiter(in) des Marktforschungsinstituts Produkt + Markt. Wir führen zurzeit eine Befragung im Auftrag des Bundesforschungsinstituts für Ernährung und Lebensmittel zum Thema „Verzehr bestimmter Lebensmittel“ durch. Das Interview dauert ca. 20 Minuten. Selbstverständlich ist die Teilnahme daran freiwillig. Bitte schenken Sie uns ein paar Minuten Ihrer Zeit und verraten uns Ihre Meinung. Herzlichen Dank im Voraus!

INTERVIEWER:	Falls danach gefragt wird:

Das Institut versichert Ihnen ausdrücklich, dass alle Ihre Angaben vertraulich behandelt und in zusammengefasster Form lediglich so ausgewertet werden, dass ein Rückschluss auf den einzelnen Befragten nicht mehr möglich ist.

Screening

Zunächst möchten wir Sie bitten, einige Angaben zu Ihrer Person zu machen, damit wir Ihre Antworten im Nachgang besser bewerten können.

S1. Sind Sie …?

männlich	☐ 1 → Quote siehe Quotenplan
weiblich	☐ 2 → Quote siehe Quotenplan

S2. Und wie alt sind Sie?

I__I__I Jahre

EDV:	Bitte in entsprechende Gruppe einordnen!

unter 18 Jahre	☐ 1 → Abbruch
18 bis 24 Jahre	☐ 2 → Quote siehe Quotenplan
25 bis 34 Jahre	☐ 3 → Quote siehe Quotenplan
35 bis 50 Jahre	☐ 4 → Quote siehe Quotenplan
51 bis 64 Jahre	☐ 5 → Quote siehe Quotenplan
65 bis 80 Jahre	☐ 6 → Quote siehe Quotenplan

S3. In welchem Bundesland leben Sie?

Schleswig-Holstein	☐ 1
Hamburg	☐ 2
Niedersachsen	☐ 3
Bremen	☐ 4
Nordrhein-Westfalen	☐ 5
Hessen	☐ 6
Rheinland-Pfalz	☐ 7
Baden-Württemberg	☐ 8
Bayern	☐ 9
Saarland	☐ 10
Berlin	☐ 11
Brandenburg	☐ 12
Mecklenburg-Vorpommern	☐ 13
Sachsen	☐ 14
Sachsen-Anhalt	☐ 15
Thüringen	☐ 16

EDV:	Bitte in entsprechende Region einordnen!

Norden (SH, HH, NDS, HB) ☐ 1 → Quote siehe Quotenplan
Osten (MVP, B, BB, SAC, SAN, THÜ) ☐ 2 → Quote siehe Quotenplan
Süden (BAY, BW) ☐ 3 → Quote siehe Quotenplan
Westen (NRW, HES, RP, SAA) ☐ 4 → Quote siehe Quotenplan

Hauptfragebogen

1. Kommen wir nun auf Mahlzeiten zu sprechen, die Sie zu Hause einnehmen. Mit „zu Hause" meinen wir im Folgenden sowohl Ihren Erst- als auch ggf. den Zweitwohnsitz.

Welche Mahlzeiten nehmen Sie üblicherweise unter der Woche von Montag bis Freitag zu Hause ein?

INTERVIEWER:	Die ersten drei Antwortmöglichkeiten vorlesen; Mehrfachnennungen möglich.

EDV:	AM 4,5 und 998 nur Einzelnennungen (AM = Antwortmöglichkeit)

Frühstück ☐ 1
Mittagessen ☐ 2
Abendessen ☐ 3

Interviewer:	Die nachfolgenden Aussagen nur vorlesen, falls keine der drei Mahlzeiten üblicherweise unter der Woche von Montag bis Freitag zu Hause eingenommen werden.

ich nehme manchmal von Montag bis Freitag eine der Mahlzeiten zu Hause ein ☐ 4 → weiter Fr. **5**
ich nehme von Montag bis Freitag keine Mahlzeit zu Hause ein ☐ 5 → weiter Fr. **5**
keine Angabe/weiß nicht ☐ 998 → weiter Fr. **5**

EDV:	Frage **2** nur stellen, wenn AM 1 in Frage **1**.

2. Mit wem nehmen Sie üblicherweise Ihr Frühstück unter der Woche von Montag bis Freitag zu Hause ein?

INTERVIEWER:	Nur eine Antwort möglich! Antwort bitte entsprechend zuordnen oder ggf. vorlesen!

allein ☐ 1
mit Personen, die mit im Haushalt leben (Partner, Kinder, Eltern, …) ☐ 2
mit Personen, die nicht mit im Haushalt leben (Freunde, Bekannte, Nachbarn, …) ☐ 3
unterschiedlich ☐ 4
keine Angabe/weiß nicht ☐ 998

EDV: Frage **3** nur stellen, wenn AM 2 in Frage **1**.

3. Mit wem nehmen Sie üblicherweise Ihr Mittagessen unter der Woche von Montag bis Freitag zu Hause ein?

INTERVIEWER: Nur eine Antwort möglich! Antwort bitte entsprechend zuordnen oder ggf. vorlesen!

allein	☐ 1
mit Personen, die mit im Haushalt leben (Partner, Kinder, Eltern, …)	☐ 2
mit Personen, die nicht mit im Haushalt leben (Freunde, Bekannte, Nachbarn, …)	☐ 3
unterschiedlich	☐ 4
keine Angabe/weiß nicht	☐ 998

EDV: Frage **4** nur stellen, wenn AM 3 in Frage **1**.

4. Mit wem nehmen Sie üblicherweise Ihr Abendessen unter der Woche von Montag bis Freitag zu Hause ein?

INTERVIEWER: Nur eine Antwort möglich! Antwort bitte entsprechend zuordnen oder ggf. vorlesen!

allein	☐ 1
mit Personen, die mit im Haushalt leben (Partner, Kinder, Eltern, …)	☐ 2
mit Personen, die nicht mit im Haushalt leben (Freunde, Bekannte, Nachbarn, …)	☐ 3
unterschiedlich	☐ 4
keine Angabe/weiß nicht	☐ 998

5. Welche Mahlzeiten nehmen Sie üblicherweise am Wochenende (Samstag und Sonntag) zu Hause ein?

INTERVIEWER: Die ersten drei Antwortmöglichkeiten vorlesen; Mehrfachnennungen möglich.

EDV: AM 4, 5, 998 nur Einzelnennungen (AM = Antwortmöglichkeit)

Frühstück	☐ 1
Mittagessen	☐ 2
Abendessen	☐ 3

Interviewer: Die nachfolgenden Aussagen nur vorlesen, falls keine der drei Mahlzeiten üblicherweise unter der Woche von Montag bis Freitag zu Hause eingenommen werden.

ich nehme manchmal am Wochenende eine der Mahlzeiten zu Hause ein	☐ 4 → weiter Fr. 9
ich nehme am Wochenende keine Mahlzeit zu Hause ein	☐ 5 → weiter Fr. 9
keine Angabe/weiß nicht	☐ 998 → weiter Fr. 9

EDV: Wenn bei Frage **1** und **5** jeweils AM 5 angekreuzt ist, dann nur Fragen 41-54, Frage 61 und Fragen 74-86 stellen.

EDV: Frage **6** nur stellen, wenn AM 1 in Frage **5**.

6. Mit wem nehmen Sie <u>üblicherweise</u> Ihr <u>Frühstück</u> zu Hause <u>am Wochenende</u> (Samstag und Sonntag) ein?

INTERVIEWER: Nur eine Antwort möglich! Antwort bitte entsprechend zuordnen oder ggf. vorlesen!

allein	☐ 1
mit Personen, die mit im Haushalt leben (Partner, Kinder, Eltern, …)	☐ 2
mit Personen, die nicht mit im Haushalt leben (Freunde, Bekannte, Nachbarn, …)	☐ 3
unterschiedlich	☐ 4
keine Angabe/weiß nicht	☐ 998

EDV: Frage **7** nur stellen, wenn AM 2 in Frage **5**.

7. Mit wem nehmen Sie <u>üblicherweise</u> Ihr <u>Mittagessen</u> zu Hause <u>am Wochenende</u> (Samstag und Sonntag) ein?

INTERVIEWER: Nur eine Antwort möglich! Antwort bitte entsprechend zuordnen oder ggf. vorlesen!

allein	☐ 1
mit Personen, die mit im Haushalt leben (Partner, Kinder, Eltern, …)	☐ 2
mit Personen, die nicht mit im Haushalt leben (Freunde, Bekannte, Nachbarn, …)	☐ 3
unterschiedlich	☐ 4
keine Angabe/weiß nicht	☐ 998

EDV: Frage **8** nur stellen, wenn AM 3 in Frage **5**.

8. Mit wem nehmen Sie <u>üblicherweise</u> Ihr <u>Abendessen</u> zu Hause <u>am Wochenende</u> (Samstag und Sonntag) ein?

INTERVIEWER: Nur eine Antwort möglich! Antwort bitte entsprechend zuordnen oder ggf. vorlesen!

allein	☐ 1
mit Personen, die mit im Haushalt leben (Partner, Kinder, Eltern, …)	☐ 2
mit Personen, die nicht mit im Haushalt leben (Freunde, Bekannte, Nachbarn, …)	☐ 3
unterschiedlich	☐ 4
keine Angabe/weiß nicht	☐ 998

Sie werden nun zum Verzehr vorgefertigter Lebensmittel und Gerichte befragt. Beachten Sie bitte, dass nur der Verzehr zu Hause gefragt ist – egal ob in der Woche oder am Wochenende. Nicht gemeint ist der Verzehr von Speisen außerhalb des Hauses z.B. in Restaurants, Kantinen, bei Bekannten, etc.

Wenn Sie jetzt einmal an die letzten 12 Monate denken, wie häufig haben Sie dann zu Hause … gegessen?

INTERVIEWER:	Skala erst erläutern („kenne ich nicht“ und „keine Angabe/weiß nicht“ nicht vorlesen!) und dann am besten die Frage erneut stellen. Auch den Text in den Klammern vorlesen!

EDV:	Fragen randomisieren!

──────────►		täglich	mehrmals pro Woche	ca. 1 Mal pro Woche	1-2 Mal pro Monat	seltener	nie	kenne ich nicht	*keine Angabe/ weiß nicht*
**9.**	fertig gemischte **Müslis**<br>(Getreideflocken oder Knuspermüsli vermischt mit Früchten, Nüssen oder Schokolade; *nicht gemeint sind Cornflakes)*	☐ 1	☐ 2	☐ 3	☐ 4	☐ 5	☐ 6	☐ 7	☐ 998
**10.**	gesüßte **Cornflakes** und ähnliches<br>(Produkte mit Schokoladen-, Honig-, Fruchtgeschmack; *nicht gemeint sind Müslis)*	☐ 1	☐ 2	☐ 3	☐ 4	☐ 5	☐ 6	☐ 7	☐ 998
**11.**	gesüßte **Joghurts**<br>(z.B. mit Zusatz von Frucht, Schoko, Nuss, Vanille oder Joghurt mit Knusper/Müsli)	☐ 1	☐ 2	☐ 3	☐ 4	☐ 5	☐ 6	☐ 7	☐ 998
**12.**	fertige, gekühlte **Desserts**<br>(z.B. Pudding, Creme, Mousse, Milchreis; nicht gemeint sind reine Joghurts und Quark mit Geschmackszutaten)	☐ 1	☐ 2	☐ 3	☐ 4	☐ 5	☐ 6	☐ 7	☐ 998

Wenn Sie jetzt einmal an die letzten 12 Monate denken, wie häufig haben Sie dann zu Hause Gerichte gegessen, die (u. a.) aus/mit … zubereitet waren?

INTERVIEWER:	Skala erläutern; „keine Angabe/weiß nicht“ und „kenne ich nicht“ nicht vorlesen! Auch den Text in den Klammern vorlesen!

EDV:	Fragen randomisieren!

──────────►		täglich	mehrmals pro Woche	ca. 1 Mal pro Woche	1-2 Mal pro Monat	seltener	nie	kenne ich nicht	*keine Angabe/ weiß nicht*
**13.**	**Nudelsoßen** aus dem Glas, der Dose oder dem Tetra Pack	☐ 1	☐ 2	☐ 3	☐ 4	☐ 5	☐ 6	☐ 7	☐ 998
**14.**	**Soßenpulver** zur Herstellung von z.B. Bratensoßen, Soße Hollandaise, Kräuter- oder Rahmsoßen	☐ 1	☐ 2	☐ 3	☐ 4	☐ 5	☐ 6	☐ 7	☐ 998

→	täglich	mehr-mals pro Woche	ca. 1 Mal pro Woche	1-2 Mal pro Monat	seltener	nie	kenne ich nicht	*keine Angabe/ weiß nicht*
**15.** **Pulver** zur Herstellung von Kartoffelpüree und/oder Kartoffelklöße/-knödel	☐ 1	☐ 2	☐ 3	☐ 4	☐ 5	☐ 6	☐ 7	☐ 998
**16.** **Brühwürfel oder -pulver** zur Herstellung von Gemüse- und Fleischbrühen	☐ 1	☐ 2	☐ 3	☐ 4	☐ 5	☐ 6	☐ 7	☐ 998
**17.** **Fix-Produkte** zur schnellen Herstellung von Fleisch-, Nudel- oder Gemüsegerichten	☐ 1	☐ 2	☐ 3	☐ 4	☐ 5	☐ 6	☐ 7	☐ 998
**18.** **Tüten-, Tassen-** oder **Instantsuppen**	☐ 1	☐ 2	☐ 3	☐ 4	☐ 5	☐ 6	☐ 7	☐ 998
**19.** **Vegane** und/oder **vegetarische Fleisch-** und **Wurstalternativen** (z.B. Bratlinge, Würstchen; *nicht gemeint sind Brotaufstriche*)	☐ 1	☐ 2	☐ 3	☐ 4	☐ 5	☐ 6	☐ 7	☐ 998

20. ***[entfällt]***

Im Folgenden möchte ich Sie zum Verzehr von <u>FERTIGGERICHTEN</u> befragen, die in Supermärkten, Discountern oder anderen Verbrauchermärkten gekauft wurden. Nicht gemeint sind Metzgereien, Wochenmärkte und Feinkostläden.
Zu den Fertiggerichten gehören die Gerichte, die vor dem Verzehr lediglich geöffnet und/oder gegart bzw. erwärmt werden müssen, wie zum Beispiel fertige Nudel-, Fleischgerichte oder Tiefkühl-Pizzen.

Wenn Sie jetzt einmal an die <u>letzten 12 Monate</u> denken, wie <u>häufig</u> haben Sie dann <u>zu Hause</u> … gegessen?

INTERVIEWER: Skala erläutern; „keine Angabe/weiß nicht“ und „kenne ich nicht“ nicht vorlesen! Auch den Text in den Klammern vorlesen!

EDV: Fragen randomisieren!

→	täglich	mehr-mals pro Woche	ca. 1 Mal pro Woche	1-2 Mal pro Monat	seltener	nie	kenne ich nicht	*keine Angabe/ weiß nicht*
**21.** tiefgekühlte **Pommes Frites**	☐ 1	☐ 2	☐ 3	☐ 4	☐ 5	☐ 6	☐ 7	☐ 998
**22.** Tiefkühl-**Pizzen**	☐ 1	☐ 2	☐ 3	☐ 4	☐ 5	☐ 6	☐ 7	☐ 998
**23.** fertige **Eintöpfe** aus Konservendose oder -glas	☐ 1	☐ 2	☐ 3	☐ 4	☐ 5	☐ 6	☐ 7	☐ 998
**24.** fertig zubereitete **Fleisch- und Wurstsalate** aus dem Kühlregal	☐ 1	☐ 2	☐ 3	☐ 4	☐ 5	☐ 6	☐ 7	☐ 998
**25.** fertig zubereitete **Kartoffelsalate** aus dem Kühlregal	☐ 1	☐ 2	☐ 3	☐ 4	☐ 5	☐ 6	☐ 7	☐ 998
**26.** fertig zubereitete **Gemüse-/ Rohkostsalate** (z.B. Krautsalat) aus dem Kühlregal	☐ 1	☐ 2	☐ 3	☐ 4	☐ 5	☐ 6	☐ 7	☐ 998
**27.** Tiefgekühlte, fertige **Nudelgerichte** mit Fleisch-, Fisch- oder Gemüsezutaten (z.B. Tiefgekühlte Lasagne)	☐ 1	☐ 2	☐ 3	☐ 4	☐ 5	☐ 6	☐ 7	☐ 998

→	täglich	mehr-mals pro Woche	ca. 1 Mal pro Woche	1-2 Mal pro Monat	seltener	nie	kenne ich nicht	*keine Angabe/ weiß nicht*
**28.** **gefüllte Teigwaren** (z.B. Maultaschen, Ravioli)	☐ 1	☐ 2	☐ 3	☐ 4	☐ 5	☐ 6	☐ 7	☐ 998
**29.** fertige Gerichte aus **Fisch oder Meeresfrüchten** aus der Tiefkühltruhe (z.B. Fischstäbchen, Schlemmerfilets)	☐ 1	☐ 2	☐ 3	☐ 4	☐ 5	☐ 6	☐ 7	☐ 998
**30.** fertige **Fleischgerichte** aus Tiefkühltruhe oder Kühlregal (z.B. Hühnerfrikassee, Gulasch, Geschnetzeltes)	☐ 1	☐ 2	☐ 3	☐ 4	☐ 5	☐ 6	☐ 7	☐ 998

31. Wenn Sie nun an alle FERTIGGERICHTE generell denken, d.h. sowohl, an die, die wir soeben besprochen haben, als auch an andere Fertiggerichte, wie häufig haben Sie diese insgesamt in den letzten 12 Monaten zu Hause gegessen?

täglich	mehrmals pro Woche	ca. 1 Mal pro Woche	1-2 Mal pro Monat	seltener	nie	*keine Angabe/ weiß nicht*
☐ 1	☐ 2	☐ 3	☐ 4	☐ 5	☐ 6	☐ 998

EDV: Wenn bei allen Fragen (**9** bis 31) jeweils AM 6, AM 7 oder AM 998 angekreuzt wird, dann direkt weiter zu Frage 41.

Welche der folgenden Aussagen treffen in den Fällen auf Sie zu, in denen Sie (persönlich) Fertigprodukte und/oder Fertiggerichte verwenden?

Ich verwende Fertigprodukte und/oder Fertiggerichte, …

INTERVIEWER: Skala erst erläutern; „keine Angabe“ nicht vorlesen!

EDV: Fragen randomisieren!

→	1 trifft voll zu	2 trifft eher zu	3 teils-teils	4 trifft eher nicht zu	5 trifft überhaupt nicht zu	*keine Angabe*
**32.** … weil ich dadurch beim Einkaufen Zeit spare<br>☐ 6 ich kaufe nicht ein	☐ 1	☐ 2	☐ 3	☐ 4	☐ 5	☐ 998
**33.** … weil sie kostengünstig sind<br>☐ 6 ich kaufe nicht ein	☐ 1	☐ 2	☐ 3	☐ 4	☐ 5	☐ 998
**34.** … weil ich dadurch bei der Zubereitung Zeit spare<br>☐ 6 ich bereite keine Mahlzeiten zu	☐ 1	☐ 2	☐ 3	☐ 4	☐ 5	☐ 998
**35.** … weil die Zubereitung von Mahlzeiten dadurch einfacher ist<br>☐ 6 ich bereite keine Mahlzeiten zu	☐ 1	☐ 2	☐ 3	☐ 4	☐ 5	☐ 998
**36.** … weil mich das an besonders stressigen Tagen entlastet<br>☐ 6 ich bereite keine Mahlzeiten zu	☐ 1	☐ 2	☐ 3	☐ 4	☐ 5	☐ 998

**37.** … weil ich nicht gerne koche<br>☐ 6 ich bereite keine Mahlzeiten zu	☐ 1	☐ 2	☐ 3	☐ 4	☐ 5	☐ 998
**38.** … weil sie mir gut schmecken	☐ 1	☐ 2	☐ 3	☐ 4	☐ 5	☐ 998
**39.** … wenn ich alleine esse	☐ 1	☐ 2	☐ 3	☐ 4	☐ 5	☐ 998
**40.** … weil sie sich gut lagern lassen bzw. damit ich einen Notvorrat habe	☐ 1	☐ 2	☐ 3	☐ 4	☐ 5	☐ 998

A) Welche der folgenden Ernährungsweisen, die ich Ihnen nun vorlese, kennen Sie?

INTERVIEWER:	Vorlesen!

EDV:	Randomisieren!

→		A) Bekannt?	B) Praktiziert?
**41.**	vegane oder vegetarische Kost	☐	☐
**42.**	überwiegend pflanzliche Kost, jedoch selten auch Fleisch (also weniger als zwei Mal pro Woche)	☐	☐
**43.**	Rohkost-Ernährung	☐	☐
**44.**	Paleo („Steinzeit“-Ernährung)	☐	☐
**45.**	Trennkost (Trennung von kohlenhydrathaltigen und eiweißhaltigen Lebensmitteln)	☐	☐
**46.**	Low-carb (kohlenhydratarme Kost)	☐	☐
**47.**	salzreduzierte Kost	☐	☐
**48.**	laktosefreie/-reduzierte Kost	☐	☐
**49.**	glutenfreie/-reduzierte Kost	☐	☐
**50.**	Reduktionsdiät (kalorienreduzierte Kost)	☐	☐
**51.**	koschere Kost (Kochen und Essen nach dem jüdischen Reinheitsgebot)	☐	☐
**52.**	halale Kost (Essen und Trinken im Islam)	☐	☐
**53.**	Sonstige Ernährungsweise, und zwar:		____________
	keine dieser / normale Ernährung		☐

B) Halten Sie sich an eine dieser gerade vorgestellten oder an eine sonstige Ernährungsweise?

INTERVIEWER:	Gemeint ist die Ernährungsweise, die aktuell überwiegend eingehalten wird.

EDV:	Nur die bekannten Ernährungsweisen einblenden.

EDV: Fr. 54 nur einblenden, wenn in Fr. 41 AM B angekreuzt wurde.

54. Wenn Sie sich vegan oder vegetarisch ernähren, in welcher Form praktizieren Sie diese Ernährungsweise?

INTERVIEWER: Erst die Antwortmöglichkeiten vorlesen; nur eine Antwort möglich.

ausschließlich pflanzliche Kost (entspricht einer veganen Kost)	☐ 1
Lakto-vegetarische Kost (schließt nur pflanzliche Lebensmittel, Milch und Milchprodukte ein)	☐ 2
Ovo-vegetarische Kost (schließt nur pflanzliche Lebensmittel und Eier ein)	☐ 3
Ovo-lakto-vegetarische Kost (schließt nur pflanzliche Lebensmittel, Eier, Milch und Milchprodukte ein)	☐ 4
Ovo-lakto-vegetarische Kost <u>mit Fisch</u> (schließt nur pflanzliche Lebensmittel, Eier, Milch, Milchprodukte, Fisch und Meeresfrüchte ein)	☐ 5
keine Angabe/weiß nicht	☐ 998

55. Wer trifft in Ihrem Haushalt <u>vorwiegend</u> die Entscheidung über die <u>Auswahl</u> der <u>einzukaufenden Lebensmittel</u>?

INTERVIEWER: Unbedingt vorlesen! Nur eine Antwort möglich.

ich <u>selbst</u> / vorwiegend <u>ich allein</u>	☐ 1
ich mit einer oder mehreren anderen im Haushalt lebenden Personen <u>zusammen</u>	☐ 2
eine oder mehrere <u>andere</u> im Haushalt lebende Personen	☐ 3
Entscheidung über die Auswahl wird von nicht im Haushalt lebenden Personen getroffen	☐ 4
Sonstige, und zwar ______________________________	|___|___|
keine Angabe	☐ 998

56. <u>Wie häufig</u> werden in Ihrem Haushalt Lebensmittel üblicherweise <u>pro Woche oder pro Monat eingekauft</u>? Gemeint sind damit nicht nur Großeinkäufe, sondern auch kleinere Einkäufe, die für den Haushalt getätigt werden und zwar von allen HH-Mitgliedern (nicht nur von Ihnen selbst).

INTERVIEWER: Nur eine Antwort möglich: Entweder pro Woche oder pro Monat.

|___|___| Mal pro Woche

|___|___| Mal pro Monat

keine Angabe/weiß nicht	☐ 998

Im Folgenden geht es um die <u>Vorbereitung UND Zubereitung</u> der <u>DREI Hauptmahlzeiten</u> (Frühstück, Mittagessen und Abendbrot) bei Ihnen <u>im Haushalt</u>.

EDV: Text bitte auf einem extra Screen einblenden!

57. Wer ist in Ihrem Haushalt vorwiegend für die Vor- und Zubereitung der drei Hauptmahlzeiten (Frühstück, Mittagessen und Abendbrot) zuständig? Zu der Zubereitung gehört neben dem Kochen auch die Vorbereitung, wie z.B. Brot schneiden, Gemüse/Obst schälen/schneiden.

INTERVIEWER:	Unbedingt vorlesen! Nur eine Antwort möglich.

ich selbst / vorwiegend ich allein	☐ 1 → weiter Fr. 58; Fr. 59+Fr. 60 überspringen
ich mit einer oder mehreren anderen im Haushalt lebenden Personen	☐ 2 → weiter Fr.58; Fr. 59+Fr. 60 überspringen
eine oder mehrere andere im Haushalt lebende Personen	☐ 3 → weiter Fr. 59
niemand, es werden keine Mahlzeiten im Haushalt zubereitet	☐ 4 → weiter Fr. 61, danach Fr. 74-86
keine Angabe	☐ 998 → weiter Fr. 61, danach Fr. 74-86

58. Wie viel Zeit nehmen Sie sich üblicherweise pro Tag für die Vor- und Zubereitung der Hauptmahlzeiten, bei denen Sie selbst dann auch mitessen? Falls mehrere der drei Hauptmahlzeiten an einem Tag zu Hause zubereitet werden, addieren Sie bitte diese Zubereitungszeiten. Bedenken Sie, dass neben dem Kochen auch die Vorbereitung (z.B. Brot schneiden, Gemüse/Obst schälen/schneiden) dazu zählen.

INTERVIEWER:	Bitte den Befragten gut stützen und ggf. mit ihm gemeinsam die Zeiten erarbeiten/berechnen. Als Hilfestellung kann direkt nach den einzelnen Hauptmahlzeiten gefragt werden. Immer in Minuten angeben und evtl. einen Taschenrechner benutzen!

	Dauer	*keine Angabe/weiß nicht*
Montag bis Freitag	|___|___| Ø Minuten pro Tag	☐ 998
Samstag und Sonntag	|___|___| Ø Minuten pro Tag	☐ 998

59. Wie viel Zeit wird üblicherweise pro Tag für die Vor- und Zubereitung der Hauptmahlzeiten von den anderen Personen im Haushalt aufgewandt, bei denen Sie selbst dann auch mitessen? Falls mehrere der drei Hauptmahlzeiten an einem Tag zu Hause zubereitet werden, addieren Sie bitte diese Zubereitungszeiten. Bedenken Sie, dass neben dem Kochen auch die Vorbereitung (z.B. Brot schneiden, Gemüse/Obst schälen/schneiden) dazu zählen.

INTERVIEWER:	Bitte den Befragten gut stützen und ggf. mit ihm gemeinsam die Zeiten erarbeiten/berechnen. Als Hilfestellung kann direkt nach den einzelnen Hauptmahlzeiten gefragt werden. Immer in Minuten angeben und evtl. einen Taschenrechner benutzen!

	Dauer	*keine Angabe/weiß nicht*
Montag bis Freitag	|___|___| Ø Minuten pro Tag	☐ 998
Samstag und Sonntag	|___|___| Ø Minuten pro Tag	☐ 998

60. Sind Sie ab und zu auch an der Vor- und Zubereitung der Hauptmahlzeiten mitbeteiligt?

ja, ich bin manchmal beteiligt	☐ 1
nein, ich bin nie beteiligt	☐ 2 → weiter Fr. 61 + Fr. 62_2; Fr. 62_1 + Fr. 63-71 überspringen
keine Angabe	☐ 998 → weiter Fr. 61; Fr. 62-71 überspringen

61. Wie gut können Sie Ihrer Meinung nach kochen?

INTERVIEWER:	Vorlesen!

sehr gut	☐ 1
gut	☐ 2
mittelmäßig	☐ 3
nicht so gut	☐ 4
gar nicht gut	☐ 5
keine Angabe/weiß nicht	☐ 998

62. 1) Wie häufig bereiten Sie selbst oder zusammen mit einer anderen Person üblicherweise aus Grundzutaten bzw. frischen Lebensmitteln eine warme Mahlzeit zu?

INTERVIEWER:	Bitte differenziert (Mo-Fr und Wochenende) abfragen und zuordnen oder ggf. vorlesen! Wie häufig an einem Tag gekocht wird, soll mit dieser Frage nicht erfasst werden! Auch tiefgefrorene Lebensmittel, die in ihrer natürlichen Form ohne weitere Zusätze eingefroren wurden, gelten als frische Zutaten.

von Montag bis Freitag (max. 5 Tage = 5 Mal)					Samstag und Sonntag (max. 2 Tage = 2 Mal)				
fast täglich (4-5 Mal)	1-3 Mal	seltener (oder: nicht jede Woche)	nie	*keine Angabe/ weiß nicht*	2 Mal	1 Mal	seltener/ nicht jedes Wochen-ende	nie	*keine Angabe/ weiß nicht*
☐ 1	☐ 2	☐ 3	☐ 4	☐ 998	☐ 1	☐ 2	☐ 3	☐ 4	☐ 998

62. 2) Wie häufig bereitet eine andere Person in Ihrem Haushalt üblicherweise aus Grundzutaten bzw. frischen Lebensmitteln eine warme Mahlzeit für Sie mit zu?

INTERVIEWER:	Bitte differenziert (Mo-Fr und Wochenende) abfragen und zuordnen oder ggf. vorlesen! Wie häufig an einem Tag gekocht wird, soll mit dieser Frage nicht erfasst werden! Auch tiefgefrorene Lebensmittel, die in ihrer natürlichen Form ohne weitere Zusätze eingefroren wurden, gelten als frische Zutaten.

von Montag bis Freitag (max. 5 Tage = 5 Mal)					Samstag und Sonntag (max. 2 Tage = 2 Mal)				
fast täglich (4-5 Mal)	1-3 Mal	seltener (oder: nicht jede Woche)	nie	*keine Angabe/ weiß nicht*	2 Mal	1 Mal	seltener/ nicht jedes Wochen-ende	nie	*keine Angabe/ weiß nicht*
☐ 1	☐ 2	☐ 3	☐ 4	☐ 998	☐ 1	☐ 2	☐ 3	☐ 4	☐ 998

EDV: Fragen 63-71 nur stellen, wenn in Frage 62 nur AM 1, 2 oder 3 gegeben wurde und nie AM 4 oder 998

Welche der folgenden Aussagen treffen auf Sie zu? Ich koche Gerichte aus frischen Zutaten, …

EDV:	Fragen randomisieren!

→	1 trifft voll zu	2 trifft eher zu	3 teils-teils	4 trifft eher nicht zu	5 trifft überhaupt nicht zu	*keine Angabe/ weiß nicht*
**63.** … weil ich das so gewohnt bin	☐ 1	☐ 2	☐ 3	☐ 4	☐ 5	☐ 998

**64.**	... auch wenn ich wenig Zeit habe	☐ 1	☐ 2	☐ 3	☐ 4	☐ 5	☐ 998
**65.**	… weil sie gesund sind	☐ 1	☐ 2	☐ 3	☐ 4	☐ 5	☐ 998
**66.**	… weil ich den Geschmack selbst bestimmen kann	☐ 1	☐ 2	☐ 3	☐ 4	☐ 5	☐ 998
**67.**	… weil ich Lebensmittelzusatzstoffe wie z.B. Geschmacksverstärker, Konservierungsstoffe, Farbstoffe vermeiden will, die in Fertigprodukten bzw. -gerichten enthalten sein könnten	☐ 1	☐ 2	☐ 3	☐ 4	☐ 5	☐ 998
**68.**	… weil ich und/oder Haushalts-mitglieder eine Allergie bzw. Nahrungsmittelunverträglichkeit habe/n und Fertigprodukte bzw. -gerichte Stoffe enthalten, die diese auslösen	☐ 1	☐ 2	☐ 3	☐ 4	☐ 5	☐ 998
**69.**	… damit ich die Menge an Fett, Salz und Zucker selbst bestimmen kann	☐ 1	☐ 2	☐ 3	☐ 4	☐ 5	☐ 998
**70.**	… wenn ich Gäste bewirte und/oder zu besonderen Anlässen	☐ 1	☐ 2	☐ 3	☐ 4	☐ 5	☐ 998
**71.**	… weil es mir Spaß bereitet/ich mich dabei entspanne	☐ 1	☐ 2	☐ 3	☐ 4	☐ 5	☐ 998

72. ***[entfällt bzw. neu zu Frage 62. 2)]***

Soziodemographie

Abschließend noch ein paar Fragen zur Statistik.

73. ***[entfällt bzw. neu zu Frage S1]***

74. Welche Staatsangehörigkeit haben Sie?

INTERVIEWER: Mehrfachnennungen möglich.

deutsch	☐ 1
übrige Europäische Union (EU), Schweiz und Großbritannien	☐ 2
sonstige Staatsangehörigkeiten	☐ 3
staatenlos	☐ 4
keine Angabe/weiß nicht	☐ 998

75. ***[entfällt bzw. neu zu Frage S2]***

76. Sind Sie in Deutschland geboren?

ja	☐ 1 → weiter Fr. 77
nein	☐ 2
keine Angabe/weiß nicht	☐ 998

76a. In welchem Land sind Sie geboren?

INTERVIEWER: Antwort bitte zuordnen!

EDV: Länderliste hinterlegen!

____ |___|___|

keine Angabe/weiß nicht ☐ 998

76b. <u>Vor wie vielen</u> Jahren sind Sie nach Deutschland gezogen?

vor |___|___| Jahren

keine Angabe/weiß nicht ☐ 998

EDV:	Nach Frage 76b weiter mit Fr. 0

77. Sind Ihre <u>Eltern</u> beide in Deutschland geboren oder hatten zum Zeitpunkt Ihrer Geburt eine deutsche Staatsangehörigkeit?

INTERVIEWER:	Vorlesen!

ja, beide	☐ 1 → weiter Fr. 0
nein, nur ein Elternteil	☐ 2 → weiter Fr. 77a
nein, beide Elternteile nicht	☐ 3 → weiter Fr. 77b
keine Angabe/weiß nicht	☐ 998 → weiter Fr. 0

77a. In welchem Land ist dieser Elternteil geboren?

INTERVIEWER:	Antwort bitte zuordnen!

EDV:	Länderliste hinterlegen!

____ |___|___|

keine Angabe/weiß nicht ☐ 998

EDV:	Nach Frage 77a weiter mit Frage 0.

77b. In welchen Ländern sind Ihre Eltern geboren?

INTERVIEWER:	Antwort bitte zuordnen!

EDV:	Länderliste hinterlegen!

1. Elternteil ____ |___|___|
2. Elternteil ____ |___|___|

keine Angabe/weiß nicht ☐ 998

78. Welchen höchsten allgemeinbildenden Schulabschluss haben Sie?

INTERVIEWER: Nur eine Antwort möglich. Vorlesen bzw. zuordnen!

Schüler/in (ohne Abschluss, allgemeinbildende Vollzeitschule)	☐ 1 → weiter Fr. 83
ohne Hauptschulabschluss (von der Schule abgegangen/ohne Volksschulabschluss)	☐ 2
Hauptschulabschluss (Volksschulabschluss/Abschluss der Polytechnischen Oberschule der DDR 8. oder 9. Klasse)	☐ 3
Realschulabschluss (Mittlere Reife/Abschluss der Polytechnischen Oberschule der DDR 10. Klasse)	☐ 4
Abschluss der Fachhochschulreife/einer Fachoberschule	☐ 5
allgemeine oder fachgebundene Hochschulreife/Abitur (Gymnasium/EOS/EOS mit Lehre)	☐ 6
anderer Schulabschluss und zwar: ______________________	|___|___|
keine Angabe	☐ 998

79. Welchen höchsten beruflichen Ausbildungsabschluss haben Sie?

INTERVIEWER: Nur eine Antwort möglich. Vorlesen bzw. zuordnen!

noch in beruflicher Ausbildung (Auszubildende/r, Student/in)	☐ 1
kein beruflicher Abschluss/nicht in beruflicher Ausbildung	☐ 2
beruflich-betriebliche Berufsausbildung (Lehre)	☐ 3
beruflich-schulische Ausbildung (Berufsfachschule/Handelsschule)	☐ 4
Ausbildung an einer Fachschule, Meister-, Technikerschule, Berufs- oder Fachakademie	☐ 5
Fachhochschulabschluss/Hochschulabschluss	☐ 6
sonstiger beruflicher Abschluss (Anlernberuf)	☐ 7
keine Angabe	☐ 998

80. Welcher Erwerbstätigkeit gehen Sie nach bzw. welche der folgenden Antworten trifft auf Sie zu? Bitte beachten Sie, dass unter Erwerbstätigkeit jede bezahlte bzw. mit einem Einkommen verbundene Tätigkeit verstanden wird.

INTERVIEWER: Nur eine Antwort möglich. Vorlesen bzw. zuordnen!

vollzeiterwerbstätig	☐ 1
teilzeiterwerbstätig	☐ 2
Altersteilzeit (unabhängig von der Phase)	☐ 3
geringfügig erwerbstätig, 450-Euro-Job, Mini Job	☐ 4
„Ein-Euro-Job“ (bei Bezug von Arbeitslosengeld 2)	☐ 5
gelegentlich oder unregelmäßig beschäftigt	☐ 6
in einer beruflichen Ausbildung/Lehre	☐ 7
in Umschulung	☐ 8

freiwilliger Wehrdienst/Bundesfreiwilligendienst oder Freiwilliges Soziales Jahr	☐ 9
Mutterschafts-, Erziehungsurlaub, Elternzeit oder sonstige Beurlaubung	☐ 10
nicht erwerbstätig (einschließlich: Schüler/-innen oder Studierende, die nicht gegen Geld arbeiten, Arbeitslose, Vorruheständler/-innen, Rentner/-innen ohne Nebenverdienst)	☐ 11 → weiter Fr. 82
keine Angabe	☐ 998

EDV: Frage 81 nur stellen, wenn Code 1, 2, 4, 5 oder 6 in Frage 80.

81. Arbeiten Sie überwiegend in Wechselschichten oder Dauernachtschicht?

ja, in Wechselschichten ohne Nachtschicht	☐ 1
ja, in Wechselschichten mit Nachtschicht	☐ 2
ja, in Dauernachtschicht	☐ 3
nein	☐ 4
Sonstiges, und zwar:	
____	|___|___|
keine Angabe	☐ 998

EDV: Nach Frage 81 direkt zu Fr. 83

82. Wenn Sie derzeit nicht erwerbstätig sind, was trifft auf Sie zu?

Student/in	☐ 1
Rentner/in, Pensionär/in, im Vorruhestand	☐ 2
arbeitslos	☐ 3
dauerhaft erwerbsunfähig	☐ 4
Hausfrau/Hausmann	☐ 5
Schüler/in der Abendschule oder des Abendgymnasiums	☐ 6
Sonstiges, und zwar: ______________________________	|___|___|
keine Angabe	☐ 998

83. Wie viele Personen – Sie selbst eingerechnet – leben in Ihrem Haushalt (inkl. Kinder)?

INTERVIEWER: Als Haushalt gilt eine Gemeinschaft von Personen, die zusammen wohnen. Zum Haushalt gehören auch Personen, die aus beruflichen Gründen vorübergehend abwesend sind. Bitte denken Sie dabei auch an alle im Haushalt lebenden Kinder.

|___|___| Person(en)

keine Angabe ☐ 998

EDV: Die Frage 84 nur stellen, wenn die Angabe in Frage 83 > 1 ist. Summenabgleich 84 zu 83!

84. Und wie viele davon sind Erwachsene und wie viele Kinder unter 18 Jahren?

INTERVIEWER: Wenn keine Kinder im Haushalt wohnen, bitte eine „0“ eintragen.

|___|___| **Erwachsene** (ab 18 Jahren) |___|___| **Kind/Kinder** (unter 18 Jahren)

keine Angabe ☐ 998

EDV: Die Frage 85 nur stellen, wenn in Frage 84 > 1 Erwachsener.

85. Leben Sie mit einer Partnerin/einem Partner zusammen?

ja ☐ 1

nein ☐ 2

keine Angabe/weiß nicht ☐ 998

86. Wie hoch ist das monatliche <u>NETTO-Einkommen</u> Ihres Haushalts insgesamt? (Ich meine damit die Summe, die sich aus Lohn, Gehalt, Einkommen aus selbständiger Tätigkeit, Rente oder Pension jeweils nach Abzug der Steuern und Sozialversicherungsbeträge ergibt. Rechnen Sie bitte auch die Einkünfte aus öffentlichen Beihilfen, Einkommen aus Vermietung, Verpachtung, Wohngeld, Kindergeld und sonstige Einkünfte hinzu.)
Ich lese Ihnen dazu nun 9 Gruppen vor. Nennen Sie mir bitte den Buchstaben der Gruppe, der sie sich zuordnen.

INTERVIEWER: Vorlesen! Die Buchstaben der Gruppe mit vorlesen! „Keine Angabe“ nicht vorlesen!

A: unter 1.500 Euro ☐ 1

B: 1.500 bis unter 2.000 Euro ☐ 2

C: 2.000 bis unter 2.500 Euro ☐ 3

D: 2.500 bis unter 3.000 Euro ☐ 4

E: 3.000 bis unter 3.500 Euro ☐ 5

F: 3.500 bis unter 4.000 Euro ☐ 6

G: 4.000 bis unter 4.500 Euro ☐ 7

H: 4.500 bis unter 5.000 Euro ☐ 8

I: 5.000 Euro und mehr ☐ 9

keine Angabe ☐ 998

Damit sind wir am Ende des Interviews angelangt. Herzlichen Dank für die Teilnahme an der Studie!
