# Supplementary material for: Sociodemographic and behavioural differences between frequent and non-frequent users of convenience food in Germany
Source: Front Nutr. 2024 Mar 22;11:1369137. doi: 10.3389/fnut.2024.1369137 (PMC10997035; doi:10.3389/fnut.2024.1369137)
Supplement: Supplementary file 1 [file Data_Sheet_1.zip › Supplementary Table 1.pdf]

Table S1. Detailed sociodemographics of the entire study sample (*N* = 3,997) of 18- to 80-year-old adults living in Germany.

Variable		Sample (*N* = 3,997, if not stated otherwise)	
		n	%
**Region**	north[1]	645	16.1
	east[2]	799	20.0
	south[3]	1,140	28.5
	west[4]	1,413	35.4
**School education**	pupils	38	1.0
	without school leaving certificate	53	1.3
	lower secondary education (9 years)	703	17.6
	higher secondary education (10 years)	1,283	32.1
	entrance qualification for universities of applied sciences (12 or 13 years)	467	11.7
	general or subject-specific university entrance qualification (12 or 13 years)	1,415	35.7
	no indication	38	1.0
**Vocational education**	in training	199	5.0
	without vocational qualification	158 [5]	4.0 [5]
	in-company vocational training	1,365	34.2
	school-based vocational training	406	10.2
	additional qualification after vocational training (e.g. at a technical college or master school)	560	14.0
	(technical) university degree	1,136	28.4
	other	83	2.1
	no indication	90	2.3
**Employment**	full-time	1,526	38.2
	part-time	602	15.1
	phased retirement	39	1.0
	marginally employed, 450-euro-job, mini job	134	3.4
	"one-euro-job" (when receiving unemployment benefits 2)	1	0.0
	occasionally or irregularly employed	24	0.6
	in vocational training	55	1.4
	in retraining	9	0.2
	voluntary military service/ federal volunteer service or voluntary social year	6	0.2
	maternity leave, parental leave or other leave of absence	44	1.1
	non-employed (incl. students, pensioners)	1,474[5]	36.9 [5]
	no indication	83	2.1
**Shift work (*n*=2,287)**	yes, in alternating shifts without night shift	253	11.1
	yes, in alternating shifts with night shift	119	5.2
	yes, in permanent night shift	77	3.4
	no	1,817	79.4
	other	11	0.5
	no indication	10	0.4
**Household size**	one-person	920	23.0
	two-person	1,634	40.9
	three-person	602	15.1
	four-person	537	13.4
	more than four-person	287	7.2
	no indication	17	0.4
**Living with a partner (*n*=2,977)**	yes	2,559	86.0
	no	406	13.6
	no indication	12	0.4
**Household net income (in €)**	less than 1,500	444	15.3
	1,500–2,000	465	16.1
	2,000–2,500	423	14.6

	2,500–3,000	385	13.3
	3,000–3,500	382	13.2
	3,500–4,000	282	9.7
	4,000–4,500	163	5.6
	4,500–5,000	118	4.1
	5,000 and more	232	8.0
	no indication	1,103	27.6
**Special diet**	yes	1,151	28.8
	vegan/vegetarian diet	196	4.9
	predominantly plant-based diet	302	7.6
	raw food diet	120	3.0
	paleo diet	21	0.5
	food combining diet	109	2.7
	low-carb diet	232	5.8
	salt-reduced diet	181	4.5
	lactose-free/reduced diet	162	4.1
	gluten-free/reduced diet	77	1.9
	reduction diet	94	2.4
	kosher diet	16	0.4
	halal diet	35	0.9
	no	2,846	71.2

[1] Schleswig Holstein, Hamburg, Lower Saxony, Bremen
[2] Mecklenburg Western Pomerania, Berlin, Brandenburg, Saxony, Saxony-Anhalt, Thuringia
[3] Bavaria, Baden-Württemberg
[4] Northrhine-Westphalia, Hesse, Rhineland, Palatinate, Saarland
[5] Incl. pupils (*n*=38, 1.0 %) who were not posed the question
